# In Vitro Screen of Lactobacilli Strains for Gastrointestinal and Vaginal Benefits

**DOI:** 10.3390/microorganisms11020329

**Published:** 2023-01-28

**Authors:** Heli Anglenius, Harri Mäkivuokko, Ilmari Ahonen, Sofia D. Forssten, Pirjo Wacklin, Jaana Mättö, Sampo Lahtinen, Liisa Lehtoranta, Arthur C. Ouwehand

**Affiliations:** 1 IFF Health and Biosciences, 02460 Kantvik, Finland; 2 Finnish Red Cross Blood Service, 00310 Helsinki, Finland; 3 Vincit Oyj, 20500 Turku, Finland

**Keywords:** *Lactobacillus*, vagina, gastrointestinal, probiotic attributes, blood group antigen, adhesion, bile tolerance, acid tolerance, hydrogen peroxide production, vaginal epithelial cells

## Abstract

Traditional probiotics comprise mainly lactic acid bacteria that are safe for human use, tolerate acid and bile, and adhere to the epithelial lining and mucosal surfaces. In this study, one hundred commercial and non-commercial strains that were isolated from human feces or vaginal samples were tested with regards to overall growth in culture media, tolerance to acid and bile, hydrogen peroxide (H_2_O_2_) production, and adhesion to vaginal epithelial cells (VECs) and to blood group antigens. As a result, various of the tested lactobacilli strains were determined to be suitable for gastrointestinal or vaginal applications. Commercial strains grew better than the newly isolated strains, but tolerance to acid was a common property among all tested strains. Tolerance to bile varied considerably between the strains. Resistance to bile and acid correlated well, as did VEC adhesion and H_2_O_2_ production, but H_2_O_2_ production was not associated with resistance to bile or acid. Except for *L. iners* strains, vaginal isolates had better overall VEC adhesion and higher H_2_O_2_ production. Species- and strain-specific differences were evident for all parameters. Rank-ordered clustering with nine clusters was used to identify strains that were suitable for gastrointestinal or vaginal health, demonstrating that the categorization of strains for targeted health indications is possible based on the parameters that were measured in this study.

## 1. Introduction

Probiotics are defined as “live microorganisms that, when administered in adequate amounts, confer a health benefit on the host” [1]. Strains of lactic acid bacteria and bifidobacteria are the most frequently used probiotics and have been recognized as safe for human consumption [2]. In addition to safety aspects, a good probiotic, if taken orally, must survive passage through the gastrointestinal tract. Thus, an effective oral probiotic should tolerate and endure the harsh acidic conditions of the stomach and bile in the small intestine [3]. Generally, probiotics confer their benefits by improving epithelial barrier function, increasing adherence to the mucosa, and competing with pathogens for sites of adherence, resulting in competitive pathogenic exclusion, the production of antimicrobial substances and bacteriocins, and modulation of the immune system [4]. Traditionally, probiotics have been used for their gastrointestinal effects and immunomodulatory functions, but increasing evidence has shown their benefits in improving the general health and well-being of a host beyond the gastrointestinal tract [5].

The vaginal microbiota has the lowest taxonomic diversity of any site in the body [6]. In most women of reproductive age, the vagina harbors the highest relative abundance of lactobacilli, comprising more than 20 species, with a predominance of only one or two species in a given sample, the most common being *Lactobacillus* (*L.*) *crispatus*, *L. iners*, *L. jensenii*, and *L. gasseri* [7,8]. In the vaginal tract, lactobacilli create a protective barrier against vaginal infections by maintaining low vaginal pH levels through the production of lactic acid and antimicrobials, such as hydrogen peroxide (H_2_O_2_) and bacteriocins [9]. Furthermore, vaginal lactobacilli inhibit pathogenesis by preventing adhesion through competitive exclusion and competition for nutrients [9]. A vaginal microbiota that is rich in lactobacilli species, such as *L. crispatus*, *L. gasseri,* and *L. jensenii,* is often associated with a lower risk of vaginal dysbiosis, i.e., bacterial vaginosis (BV) and other vaginal infections, as well as healthy reproduction [8,10]. However, not all lactobacilli strains confer these benefits, and some benefits are strain-dependent, rendering them effective only for certain disease conditions or pathogens [9,11].

The so-called secretors—individuals who express fucosyltransferase 2 (FUT2)—express blood group determinants (ABO blood group antigens) on mucus and mucosal cells in the intestine and vagina. These antigens are not present in non-secretor individuals who lack FUT2 [12]. The composition of fecal bifidobacteria differs substantially between secretors and non-secretors, the latter of whom harbor lower amounts and fewer species in feces than secretors [13]. Secretor status and FUT2 polymorphisms have been suggested to affect the composition of the gut microbiota [14]; thus, the characteristics of adhesion to specific blood group antigens can be used to develop personalized adhesive probiotics, i.e., strains that interact with particular blood group determinants.

In this study, we screened 100 *Lactobacillus sensu lato* strains with regard to their tolerance to acid and bile, as well as their ability to grow in de Man–Rogosa–Sharpe (MRS) medium. We also measured parameters that are related to vaginal health, such as H_2_O_2_ and adhesion to vaginal epithelial cells (VECs), as well as studied their adherence to blood group antigens to determine strain- and host-specific differences in adhesion. By statistical clustering, we found that it is possible to classify lactobacilli strains as “classical” gastrointestinal probiotics with good tolerance to acid and bile or as probiotics for vaginal health with good vaginal cell adhesion and H_2_O_2_ production. The strains also showed strain-specific differences, highlighting the uniqueness of each strain.

## 2. Materials and Methods

### 2.1. Bacterial Strains

A total of 100 strains were screened (Table 1), most of which were *Lacticaseibacillus rhamnosus* (20 strains), followed by *Lactobacillus acidophilus* (15), *Limosilactobacillus fermentum* (10), *L. crispatus* (9), *Lacticaseibacillus paracasei* (7), and *L. jensenii* and *L. gasseri* (6 each). The remaining strains had 5 or fewer members each.

Fifty isolates were sourced from the fecal samples of healthy Finnish adult volunteers (ethical permission 229/E0/07, Helsinki University Hospital EC). A total of 35 strains were obtained from the Danisco Global Culture Collection (DGCC, Niebüll, Germany), containing commercialized (15 strains) and non-commercial strains (20 strains) with dairy, plant, fecal, or unknown origin, all of which were arbitrarily selected based on their lactobacilli status.

A total of 15 strains were isolated from the vaginal tract of asymptomatic healthy Swedish women according to Amsel criteria with a pH of 4.1 (kindly donated by Dr. Inger Mattsby-Baltzer, University of Gothenburg, Gothenburg, Sweden) (Table 1): 5 *L. crispatus*, 5 *L. iners*, and 5 *L. jensenii*. Of the 5 *L. iners* strains, 2 failed to grow under laboratory conditions and were therefore omitted from the data analysis. In total, 98 strains were tested for general growth, tolerance to acid and bile, VEC adhesion, H_2_O_2_ production, and adherence to blood group antigens.

The comparator strains were as follows: *L. rhamnosus* GG (VTT E-96666, VTT Culture Collection, Espoo, Finland) for acid and bile tolerance; *L. jensenii* DSM20557 (Deutsche Sammlung von Mikroorganismen und Zellkulturen GmbH (DSMZ), Braunschweig, Germany) for H_2_O_2_ production; and *L. crispatus* LMG18199 (JCM8778) (Laboratorium voor Microbiologie, Universiteit Gent, Ghent, Belgium), which is known to adhere well to the A antigen [15], for adhesion to the A, B, and H antigens. In a screen, *L. jensenii* LX11796 adhered well to VECs and was therefore selected as a comparator strain for the VEC adhesion assay.

### 2.2. Acid and Bile Tolerance

The tolerance of all strains to acid was tested at pH 2.5 and pH 3.5 for 1.5 h, and tolerance to bile was measured in the presence of 0.9% and 0.3% oxgall (Difco, Fisher Scientific, Vantaa, Finland) for 24 h [16]. In these assays, *L. rhamnosus* GG was included for comparison based on its ability to survive and proliferate at gastric pH and in bile-containing medium [17].

Briefly, the strains were cultured in duplicate in MRS broth under anaerobic conditions at 37 °C for 18 h. Next, the bacteria were collected by centrifugation at 2800× *g* for 10 min. The pelleted cells were washed twice with 10 mL phosphate-buffered saline (PBS 0.01 mol/L; pH 7.2, Thermo Fisher Scientific, Waltham, MA, USA) and resuspended in PBS to an optical density at 600 nm (OD_600_) of 1 (equaling approximately 1 × 10^8^ colony-forming units (CFU)/mL).

Acid tolerance was tested by incubating cells in PBS at pH 2.5, 3.5, and 7.2 for 90 min at 37 °C. Tenfold dilution series were grown on MRS agar, and colonies were counted after a 48 h incubation under anaerobic conditions at 37 °C. The results are expressed as log reduction in growth in CFU at pH 2.5 and 3.5 versus pH 7.2.

Bile tolerance was tested by incubating diluted (1:10) strain cultures in MRS broth that contained 0%, 0.3%, or 0.9% oxgall at 37 °C for 24 h under anaerobic conditions. Growth was measured before and after incubation as OD_595_ on a Multiskan RC (Labsystems Oy, Vantaa, Finland). The results are expressed as % growth (OD_600_) in MRS with 0.9% or 0.3% oxgall versus without bile.

### 2.3. Hydrogen Peroxide Production

The strains were cultured overnight in MRS broth under microaerophilic conditions at 37 °C. OD_600_ absorbance was then measured on a Multiskan RC, averaging 2.2 (range: 1.8 to 2.4). For the H_2_O_2_ assay, 1 mL of the culture was inoculated in 10 mL MRS broth and incubated under aerobic conditions for 3 h at 37 °C to induce H_2_O_2_ production. A 50 μL sample was taken from the aerated culture at the start of the incubation (0 h) and after 1.5 and 3 h. H_2_O_2_ in the 50 μL sample was measured using a hydrogen peroxide fluorometric detection kit (AH diagnostics Oy, Helsinki, Finland) according to the manufacturer’s instructions. Briefly, 50 μL of reaction cocktail was added to the 50 μL sample and incubated for 10 min in the dark at room temperature, after which the fluorescence was measured on a Wallac Viktor^2^ 1420 multilabel counter (Perkin Elmer, Turku, Finland). Results are expressed as H_2_O_2_ production after 0 h, 1.5 h, and 3 h fermentation versus the reference strain, *L. jensenii* DSM20557. All measurements were performed in duplicate.

### 2.4. Adhesion to A, B, and H Antigens

The blood group antigens A, B, and H (H antigen corresponding to blood group O phenotype) (Elicityl, Crolles, France) were suspended in PBS at pH 7.2 and biotinylated. The strains were cultured on MRS plates under anaerobic conditions at 37 °C for 48–72 h. A single colony was used to reinoculate 10 mL MRS broth and cultured overnight under anaerobic conditions at 37 °C. The cells were washed twice with 10 mL PBS (pH 7.2) and resuspended in PBS to an OD_600_ of 1 (approximately 1 × 10^8^ CFU/mL).

Then, 1 mL of bacterial sample and 1 mL 10 µg/mL biotinylated antigen solution were mixed and incubated with slow shaking for 30 min at room temperature, and 100 µL of this mixture was transferred to Delfia streptavidin-coated 96-well plates (Perkin Elmer). The plates were washed twice with 200 µL PBS at pH 7.2 three times with SuperBlock (twice with 200 µL and once with 100 µL) (ThermoFisher Scientific, Pierce™, Waltham, MA, USA) and once with 200 µL sterile water. The plates were then incubated for 30 min at room temperature with slow agitation, after which each well was washed 3 times with 200 µL sterile water for 5 min each. To detect the attached bacteria, 200 µL Syto9 dye (diluted 1:6) (Invitrogen) was added to each well and incubated for 15 min in the dark. The intensity of the dye was measured on a Wallac Viktor^2^ 1420 multilabel counter; all measurements were performed in quadruplicate, and the results were repeated at least twice.

### 2.5. Adhesion to VECs

Primary VECs were obtained from healthy adult donors (age not specified) (CelProgen, San Pedro, CA, USA), maintained at 37 °C in a 5% CO_2_ atmosphere, and expanded in human vaginal epithelial expansion ECM T75 flasks (CelProgen) in human VEC growth media with serum (CelProgen). The cells were used in the adhesion assays at the earliest passage possible.

For the adhesion assays, 150,000 VECs were seeded in human vaginal epithelial cell culture ECM 24-well plates (CelProgen). After overnight incubation at 37 °C in 5% CO_2_, the cells were washed 2 times with Dulbecco’s Modified Eagle Medium (DMEM, Thermo Fisher Scientific) without supplements. Radioactively labeled bacteria (see below) were then applied to the cells and incubated for 1 h to attach. The cells were washed 4 times with PBS at pH 7.2 to remove unattached bacteria, after which 100 µL DMEM and 1 mL Optiphase Supermix (Perkin Elmer) were pipetted onto the cells.

The bacterial strains were labeled radioactively by transferring 1% inoculation from overnight cultures into 1.5 mL MRS with 10 µL methyl-1.2 [^3^H]thymidine (4.4 TBq/mmol) (Perkin Elmer) and anaerobically cultured overnight at 37 °C. The next day, the bacteria were collected by centrifugation at 2800× *g* for 5 min, and the bacterial pellet was suspended in PBS and washed twice with PBS. The bacterial quantity was determined by flow cytometry as previously described [18]. Then, 10 bacteria per vaginal cell were centrifuged and diluted with DMEM (Thermo Fisher Scientific™). The number of bacteria in the adhesion experiments was determined in an initial optimization screen with 7 strains (data not shown).

The radioactivity was counted on a 1450 Microbeta Trilux liquid scintillation and luminescence counter (Perkin Elmer). The adhesion for each bacterium was calculated as follows:

(1) $$\%Adhesion=\frac{Sample\;value-control\;1}{control\;2-control\;3}\times 100$$

where:

- Control 1 contained VECs but no bacteria;
- Control 2 contained the same number of bacteria but no VECs, which represents the maximum radioactive count that can be obtained from a sample;
- Control 3 comprised empty wells without bacteria or VECs.

Because *L. jensenii* LX11796 (Table 1) showed good adhesion, with 5.9% (±2.7) of bacteria adhering to VECs, it was selected as a comparator strain. Thus, relative adhesion was calculated as the ratio of the % adhesion of the sample to the mean % adhesion value of the comparator.

### 2.6. Statistical Methods

Species were compared using a Welch t-test (row vs. column). FDR adjustment was performed using the Benjamini–Hochberg procedure (supplemental Tables S1–S6). In these analyses, species with fewer than 3 strains (*Lactococcus lactis*, *L. amylovorus*, *L. casei*, *L. curvatus*, *L. delbrueckii*, *L. helveticus*, *L. johnsonii*, *L. mucosae*, *L. reuteri*, *L. ruminis*, and *L. salivarius*) were omitted from the analysis.

Spearman correlation was performed to assess the statistical dependence between measurement pairs. The statistical significance of Spearman correlation coefficients was calculated using GraphPad Prism, version 9.2.0 (GraphPad Software, La Jolla, CA, USA), and *p* values < 0.05 were considered significant.

The adhesion data for antigens A, B, and C were standardized separately to 0 mean and unit variance for descriptive purposes.

Part of the analysis was performed using rank-transformed data that were derived by rank ordering the strains based on a measurement, then by replacing the measured value by the rank of the strain such that the best-performing strain was assigned a value of 1 and the worst-performing strain was assigned a value of 98. As not all strains produced H_2_O_2_, the maximum value of H_2_O_2_ production either at 0 h, 1.5 h, or 3 h was taken for ranking. The benefit of this approach includes the ability to compare and visualize the measurements. The strains were clustered by Euclidean distance in the rank-transformed data using Ward’s method [19]. The resulting tree was pruned into 9 clusters for descriptive purposes.

The statistical significance of comparisons between strain groups using ranked data was calculated by the Kruskal–Wallis rank sum test [20] (Supplementary Materials Figures S7–S10).

Statistical analyses were performed in R (version 4.0.3) and with GraphPad Prism (version 9.2.0).

## 3. Results

### 3.1. Growth

**Figure 1:**
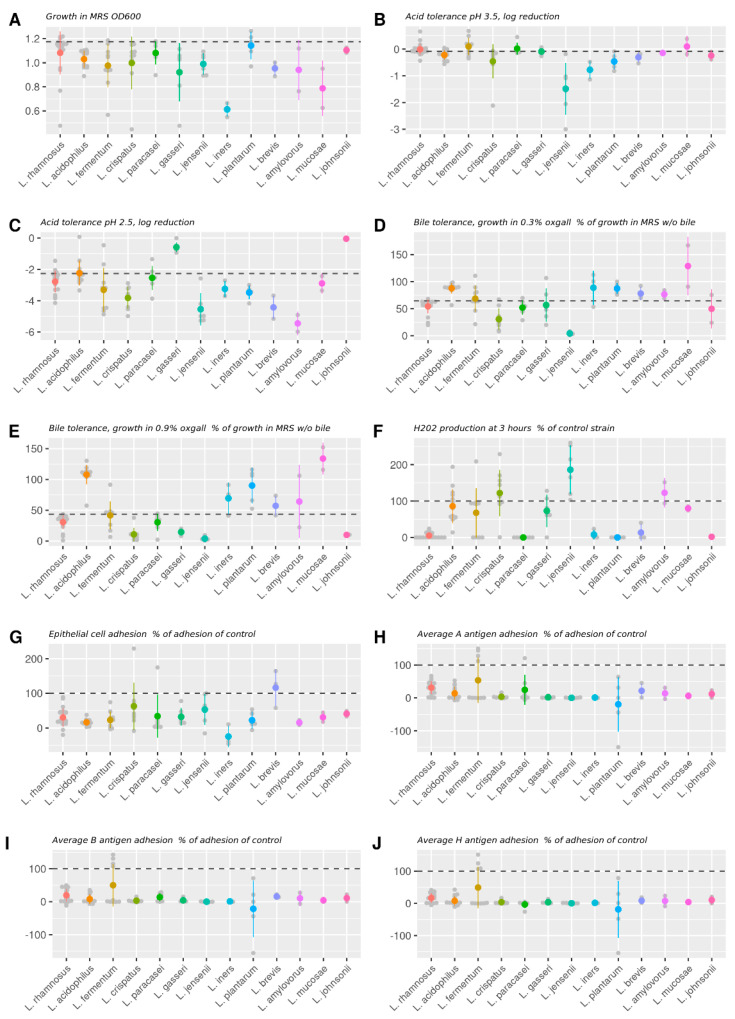
Probiotic characteristics: growth, acid, and bile tolerance, as well as H_2_O_2_ production and adhesion characteristics at the species level. (**A**) MRS growth, OD600; (**B**) acid tolerance in pH 3.5, log reduction; (**C**) acid tolerance in pH 2.5, log reduction; (**D**) bile tolerance in 0.3% oxgall, % growth in MRS without (w/o) bile; (**E**) bile tolerance in 0.9% oxgall, % growth in MRS w/o bile; (**F**) H_2_O_2_ production expressed as H_2_O_2_ production in relation to comparator strain *L. jensenii* DSM 20557 after 3 h; (**G**) vaginal epithelial cell adhesion, percentage (%) of adhesion of *L. jensenii* LX11796; (**H**) average A-antigen adhesion, % of adhesion of *L. crispatus* LMG 18204; (**I**) average B-antigen adhesion, % of adhesion of *L. crispatus* LMG 18204; and (**J**) average H-antigen adhesion, % of adhesion of *L. crispatus* LMG 18204. Mean ± SD values from species with two or more strains in the panel, as well as the individual values of each strain of a particular species, are shown. The acid tolerance data at pH 3.5 in Figure 1B are missing from *L. acidophilus* LA11897, *L. amylovorus* LX11898, *L. paracasei* LC11896, and *L. ruminis* LX11894. Furthermore, *L. paracasei* and *L. plantarum* species are omitted from Figure 1F, as they did not produce H_2_O_2_. Two *L. iners* strains from the panel did not grow and were omitted from the analysis. The dashed line in (**A**–**E**) indicates the value obtained from the control strain *L. rhamnosus* GG. The dashed line in (**F**) indicates the value obtained from the comparator strain *L. jensenii* DSM 20557, in Figure 1G the comparator strain *L. jensenii* LX11796 and in (**H**–**J**) the adhesion of comparator strain *L. crispatus* LMG18199, that is known to adhere well to the A blood group antigen.

The ability to grow in MRS broth was tested for the entire panel of strains. At the species level, most species grew well in MRS, although high variation was observed in certain species (Figure 1A). The only species that did not grow in MRS were two strains of *L. iners* (Li25-34 and Li22-26), which were therefore excluded from further studies. In addition, according to optical density measurements, the other *L. iners* strains grew more poorly than the other species, with the exception of *L. gasseri* (Table S1).

At the strain level (Supplementary Materials Figure S1), *L. rhamnosus* LX11888 showed the highest variation in growth amongst individual strains. The 10 worst-growing strains in MRS were the 3 *L. iners* strains, *L. amylovorus* LX11898, *L. mucosae* LX11854, *Latilactobacillus curvatus* 360, *L. fermentum* 508, *L. rhamnosus* LX11870, *L. gasseri* LG11876, and *L. crispatus* LX11211. None of the poor performers was a commercial strain. The 10 best growers in MRS were the commercial strains *L. salivarius* Ls-33, *L. plantarum* Lp-115, *L. rhamnosus* HN001, and *Lacticaseibacillus casei* Lc-11, in addition to the non-commercial strains *L. plantarum* LX11878 and LX11861, *L. rhamnosus* 1704 and LR1049, *L. fermentum* LX1312, and *Lacticaseibacillus paracasei* LX11858.

### 3.2. Acid Tolerance

Acid tolerance of the 98 strains in MRS was tested by growing them at pH 2.5 and pH 3.5; the logarithmic reduction in growth was normalized to the growth at pH 7.2 (Figure 1B,C). Acid tolerance of *L. rhamnosus* GG was used for comparison. The acid tolerance at pH 2.5 correlated positively with that at pH 3.5 (pairwise Spearman correlation, *r* = 0.2782 and *p* = 0.0066 (data not shown)).

The acid tolerance varied widely between species, but many tolerated the milder acidic conditions at pH 3.5 well (Figure 1B), particularly *L. rhamnosus, L. acidophilus, L. fermentum, L. paracasei*, *L. gasseri*, and *L. crispatus* (with the exception of one strain). *L. jensenii* had the highest variation among species, with lower tolerance to acid, and did not significantly differ from *L. fermentum* (Table S2). The other significant differences were between *L. acidophilus* and *L. rhamnosus*, as well as between *L. fermentum* and *L. plantarum* (Table S2), which indicates that the various species tolerated mild acid quite well.

Eighty-seven strains showed high survival in acid at pH 3.5 (less than 0.5 log reduction) (Supplementary Materials Figure S2A), with an average log reduction of 0.26 (SD ± 0.57) compared to *L. rhamnosus* GG (−0.08 ± 0.23). Viability at pH 3.5 decreased by more than 2 logs for only four strains: *L. jensenii* LX11794, *L. crispatus* LX11211, *L. curvatus* 360, and *L. jensenii* LX12217.

At pH 2.5 (Figure 1C), all six *L. gasseri* strains were among those that tolerated acid the best (log reduction < 1). Indeed, *L. gasseri* was significantly more tolerant than all other species (Table S3). *L. acidophilus* species tolerated acid at pH 2.5 quite well and were significantly more tolerant than *L. crispatus*, *L. plantarum*, and *L. jensenii* (Table S3). Similarly, *L. paracasei* and *L. rhamnosus* were more tolerant than *L. crispatus* and *L. jensenii* (Table S3). Visually, *L. amylovorus* and *L. brevis* strains also performed poorly, and *L. johnsonii* was the best-performing in terms of acid tolerance, although the number of strains was too low for proper statistical analysis (Figure 1C and Table S3).

At pH 2.5, viability decreased by more than 2 logs in 83 strains (Supplementary Materials Figure S2B), and the tolerance varied more widely than at pH 3.5; the average reduction in viability was 2.96 (SD ± 1.34). Compared with *L. rhamnosus* GG, which underwent a log reduction of 2.27 (SD ± 0.35), 23 strains were more tolerant to acid at pH 2.5 (Supplementary Materials Figure S2B). A log reduction of more than 5 was noted in *L. acidophilus* LA0893, *L. jensenii* LX12216, *L. crispatus* LX1307, *L. jensenii* LX11796, *L. brevis* LX11860, *L. jensenii* LX11795, *L. jensenii* LX12217, and *L. amylovorus* LX11898.

### 3.3. Bile Tolerance

To test the bile tolerance of the strains, their growth in MRS was compared with and without bile using *L. rhamnosus* GG as the comparator. The tolerance to 0.3% and 0.9% bile correlated positively (pairwise Spearman correlation, *r* = 0.858 and *p* < 0.0001 (data not shown)). Furthermore, in the Spearman correlation analysis, the tolerance to acid at pH 2.5 correlated positively to 0.3% and 0.9% bile tolerance (*r* = 0.223, *p* = 0.027 and *r* = 0.216, *p* = 0.033, respectively (data not shown)).

At the species level, the variation was much higher at milder (0.3%) (Figure 1D) versus 0.9% bile (Figure 1E), and the bacteria generally tolerated 0.3% bile well and similarly to the comparator strain. The weakest-performing species in 0.3% bile (Figure 1D) was *L. jensenii*, with significantly worse performance than the rest of the species, with the exception of *L. iners* (Table S4). Likewise, *L. crispatus* grew poorly in 0.3% bile and performed significantly worse than every other species, except *L. paracasei*, *L. gasseri,* and *L. iners* (Table S4). The best-performing species in 0.3% bile was *L. mucosae*, with its two strains, but only visually. Statistically, the best-performing species was *L. acidophilus*, which performed better than *L. paracasei*, *L. rhamnosus*, *L. crispatus,* and *L. jensenii* (Figure 1D and Table S4).

At the strain level, in 0.3% bile (Supplementary Materials Figure S3A), 40 strains performed better than *L. rhamnosus* GG, and survival in 31 strains decreased by more than 50%. Twelve strains had a survival rate of 10% or lower.

In 0.9% bile (Figure 1E), two distinct groups appeared: one with better tolerance, comprising *L. acidophilus*, *L. iners, L. plantarum*, *L. brevis, L. amylovorus*, and *L. mucosae*, exceeding the survival of *L. rhamnosus* GG; and one of less tolerant species, with survival rates at or below that of the comparator, including *L. rhamnosus, L. fermentum, L. crispatus, L. paracasei*, *L. gasseri*, *L. jensenii*, and *L. johnsonii*. Indeed, *L. acidophilus* species performed significantly better than any other species except *L. brevis*, *L. plantarum,* and *L. iners* (Table S5), and similarly, *L. plantarum* performed better than any other species except for *L. acidophilus*, *L. brevis*, *L. fermentum,* and *L. iners* (Table S5).

In 0.9% bile, 36 strains performed better than *L. rhamnosus* GG, and survival in 69 strains decreased by more than 50%. A total of 22 strains had survival rates of 10% or less (Supplementary Materials Figure S3B).

### 3.4. Hydrogen Peroxide Production

H_2_O_2_ production by each strain was measured after 1.5 h and 3 h of incubation, expressed as a percentage versus the comparator *L. jensenii* DSM 20557 [21]. H_2_O_2_ production correlated strongly between the two time points (pairwise Spearman correlation, *r* = 0.788, *p* < 0.0001 (data not shown)). Of the 98 tested strains, only 50 produced H_2_O_2_ after 1.5 h; therefore, only the H_2_O_2_ production after 3 h was more closely investigated at the species level. Notably, H_2_O_2_ production at 1.5 h correlated negatively with acid tolerance at pH 2.5 (*r* = −0.230, *p* = 0.023 (data not shown)) and pH 3.5 (*r* = −0.312, *p* = 0.002 (data not shown)) and with bile tolerance at 0.3% (*r* = −0.229, *p* = 0.023 (data not shown)) and 0.9% oxgall (*r* = −0.259, *p* = 0.01 (data not shown)) and positively with VEC adhesion (*r* = 0.201, *p* = 0.048 (data not shown)). Similarly, H_2_O_2_ production at 3 h had an inverse relationship with acid tolerance at pH 2.5 (*r* = −0.227, *p* = 0.025 (data not shown)) and pH 3.5 (*r* = −0.304, *p* = 0.003 (data not shown)) and with bile tolerance at 0.3% (*r* = −0.207, *p* = 0.041 (data not shown)).

H_2_O_2_ production varied highly within species, indicating strain-specific differences. All *L. acidophilus*, *L. jensenii*, *L. gasseri* (excluding *L. gasseri* Lg-36), and *L. crispatus* strains (excluding *L. crispatus* LX11211) produced H_2_O_2_ at 3 h, whereas *L. rhamnosus*, *L. johnsonii*, *L. paracasei*, and *L. plantarum* strains generated little, if any (Figure 1F). *L. fermentum* strains were variable in production, as some strains produced relatively high amounts, whereas some did not produce at all (Figure 1F). Furthermore, vaginal isolates of *L. iners* strains synthesized limited H_2_O_2_ compared to other vaginal isolates. *L. crispatus* and *L. jensenii. L. jensenii* produced the highest mean concentration of H_2_O_2_ at 3 h, which was significantly better than almost every other species, except for *L. acidophilus* and *L. crispatus* (Figure 1F and Table S6). Additionally, apart from low-producing outliers, *L. crispatus* generated substantial amounts of H_2_O_2_ and performed better than every other species except *L. acidophilus*, *L. fermentum*, *L. gasseri,* and *L. jensenii* (Table S6).

The number of strains that produced H_2_O_2_ increased from 50 to 62 after 3 h incubation (Supplementary Materials Figure S4). H_2_O_2_ production was undetectable in 36 strains. A total of 23 strains performed better than the comparator at 3 h: 6 *L. jensenii*, 5 *L. acidophilus*, 5 *L. crispatus*, 5 *L. gasseri*, *Ligilactobacillus ruminis* LX11894, *L. amylovorus* LX11898, *L. delbrueckii* subsp. *bulgaricus* Lb-64, *Lactococcus lactis* Ll-23, and *L. fermentum* LX11865 strains (Supplementary Materials Figure S4). Almost all vaginal isolates produced H_2_O_2_ and were among the top 23 strains performing better than *L. jensenii* DSM 20557, with the exception of *L. crispatus* LX11211 and all *L. iners* strains, which produced limited H_2_O_2_, and *L. crispatus* LX12212, which generated 97% of the H_2_O_2_ levels produced by the control strain.

### 3.5. Adhesion to VECs

The adhesion of bacteria to primary VECs was measured by applying them to VECs at a ratio of 10 tritium-labeled lactobacilli to 1 VEC and by incubating them for 1 h, after which adhered bacteria were counted after washing away the unadhered bacteria. To compare the results between experiments, adhesion in each assay was normalized to that of the comparator strain, *L. jensenii* LX11796. The adhesion to VECs correlated positively with H_2_O_2_ production at 1.5 h (Spearman r = 0.201, *p* = 0.048 (data not shown)) and negatively with tolerance to 0.3% bile (Spearman r = −0.222, *p* = 0.029 (data not shown)).

At the species level (Figure 1G), the adhesion was quite uniform. *L. brevis*, with its three strains, seemed to adhere the best to the cells, whereas *L. iners* represented the least adherent species. However, when analyzed statistically, no statistical differences were detected among strains (Table S7).

At the strain level, the bacteria exhibited wide range of adhesion (Supplementary Materials Figure S5). The top 15 strains that adhered the best to VECs included 3 *L. rhamnosus* strains (LR1049, LX11875, and LX11881), 3 *L. jensenii* strains (LX11795, 911, and LX11796), and 3 *L. crispatus* strains (LX1220, LX11797, and LX11799). *L. reuteri* 1753, two *L. brevis* strains (LX11860 and LX11864), *L. paracasei* LX11887, and *L. crispatus* LX11799 adhered better than or equal to the comparator strain. As the comparator strain in the screen was not a commercial or type strain, it was difficult to determine how well the bacteria adhered compared with a benchmark. However, the strain utilized as comparator was adhered better than the commercial strains in the panel.

### 3.6. Adhesion to Blood Group Antigens A, B, and H

The adhesion of the strains to blood group antigens A, B, and H was measured by in vitro assay using biotinylated antigens and compared with the comparator strain, *L. crispatus* LMG18199, which adheres well to the A antigen [22]. The specificity for the antigens was low, and most strains adhered similarly to all tested antigens. The adhesiveness also correlated strongly between antigens (pairwise Spearman correlation *r* = 0. 819 between adhesion to A and B, *r* = 0.700 between A and H, and *r* = 0.788 between B and H; *p* < 0.0001 for all (data not shown)). These values did not correlate well with the other measured parameters in the Spearman correlation analysis.

At the species level, all species adhered to the antigens at similar levels (Figure 1H–J). *L. fermentum*, *L. paracasei*, and *L. plantarum* species showed the highest variation between strains; therefore, some strains from these species displayed differences in adherence to the antigens. However, statistical significance was only observed between *L. rhamnosus* and *L. crispatus*, *L. gasserii*, *L. jensenii*, and *L. iners*, with higher adhesion to all blood type antigens (Tables S8–S10). Furthermore, *L. rhamnosus* was significantly more adhesive compared to *L. paracasei* in the case of antigen H adhesion (Table S10).

Some strains showed high variation in their adherence to antigens, whereas others did not adhere or did so to a limited extent (Supplementary Materials Figure S6A–C). Five strains showed better adhesion to the A antigen than the comparator strain: *L. paracasei* LX11858, *L. fermentum* LX11866, *L. fermentum* LX11865, *L. fermentum* LX11852, and *L. fermentum* LX11853 (Supplementary Materials Figure S6A), of which the latter four also adhered to B and H antigens better than the comparator strain (Supplementary Materials Figure S6B,C).

**Figure 2:**
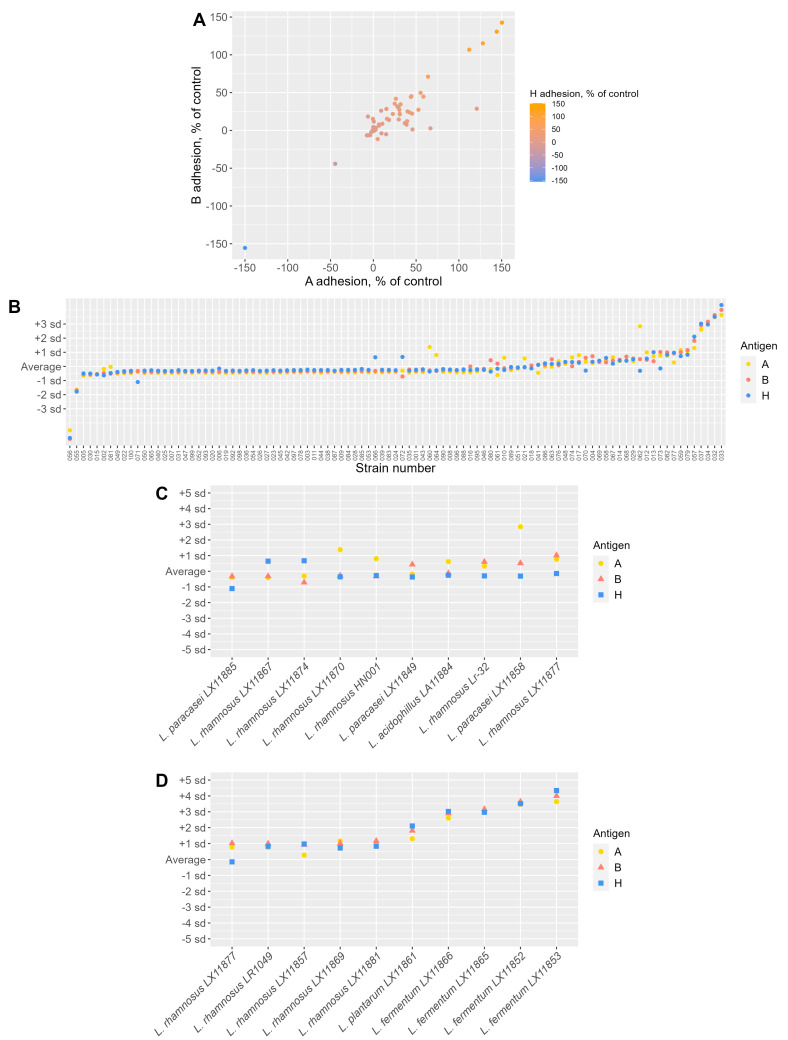
(**A**) Correlation of the adherence to A, B, and H antigens. (**B**) Standardized measures of antigen adhesion. (**C**) The top 10 strains with the greatest standard deviation (SD > 0.58). (**D**) Strains with the highest median adhesion to the three antigens.

Plotted together (Figure 2A), the adhesion values correlate well between antigens, indicating that a strain with strong A-antigen adhesion is also likely to have strong B- and H- antigen adhesion, as indicated above. When adhesion values were standardized for each antigen to a comparable scale by setting their standard deviations to 1, the adhesion was remarkably similar for each strain (Figure 2B). Several exceptions were observed, particularly when the standardized adhesions within each strain were examined. The 10 strains with the highest standard deviations (SD > 0.58) and, most likely, the highest specificity for a certain blood group antigen (Figure 2C) were 6 *L. rhamnosus* strains (LX11870, HN001, LX11867, Lr-32, LX11874, and LX11877), 3 *L. paracasei* strains (LX11858, LX11885, and LX11849), and *L. acidophilus* LA11884. In contrast, the 10 least-varying strains (SD < 0.03) and, most likely, adhering similarly to all blood group antigens were the 2 strains of *L. amylovorus* (LX11891 and LX11898), 2 strains of *L. gasseri* (LG11876, LG11895), 2 strains of *L. mucosae* (LX11893 and LX11854), *L. helveticus* LH0138, *L. acidophilus* LX11850, *L. crispatus* LX11797, and *L. rhamnosus* LX11851 (data not shown). The 10 strains with the highest median adhesion values to the 3 antigens (Figure 2D) were 5 *L. rhamnosus* strains (LX11877, LR1049, LX11857, LX11869, and LX11881), 4 *L. fermentum* strains (LX11866, LX11865, LX11852, and LX11853), and *L. plantarum* LX11861.

#### Differences in Commercial Strains and Vaginal Isolates

Next, the data were rank-ordered and analyzed as groups (fecal versus non-fecal isolates, commercial versus non-commercial, and vaginal versus non-vaginal). In the rank ordering, the best-performing probiotic was assigned a value of 1, increasing in value for each strain to 98, with the exception of ranking based on H_2_O_2_ production (see Section 2.3). The rank-ordered data were also correlated to gastrointestinal and vaginal parameters, and in each correlation analysis, the 10 best-performing probiotics were identified. Generally, with regard to gastrointestinal probiotic characteristics, fecal lactobacilli were the top performers, whereas vaginal isolates predominated when vaginal probiotic characteristics (H_2_O_2_ production and adhesion to vaginal epithelial cells) were analyzed.

Compared with non-fecal strains (Supplementary Materials Figure S7), fecal lactobacilli showed better tolerance to 0.3% and 0.9% bile (*p* = 0.002 and *p* < 0.001, respectively) and greater adhesion to the A (*p* < 0.001), B (*p* = 0.004), and H antigens (*p* = 0.006). No other significant parameters were noted.

When commercial strains were compared to non-commercial strains, the 15 studied commercial strains grew better in MRS (*p* = 0.009). Interestingly, the current commercial strains showed less adhesion to VECs than the non-commercial strains (*p* = 0.030) (Supplementary Materials Figure S8A).

We also projected the rank-ordered data in two dimensions to determine the best-performing strains in each projection. When the rank-ordered data for general growth in MRS and acid tolerance at pH 2.5 were projected, three commercial strains—*L. casei* Lc-11, *L. rhamnosus* HN001, and *L. rhamnosus* Lr-32—were among the 10 best-performing strains (Supplementary Materials Figure S8B). *L. rhamnosus* strains were generally well represented in growth versus acid tolerance, contributing 6 of the top 10 strains (HN001, Lr-32, LX11882, LX11877, LX11879, and LX11881), with *L. gasseri* 811, *L. johnsonii* LG0884, *L. casei* Lc-11, and *L. acidophilus* LA11883 constituting the remainder.

Similarly, tolerance to 0.3% and 0.9% bile was projected to acid tolerance at pH 3.5 and pH 2.5 (data not shown; Supplementary Materials Figure S9A). Under the milder conditions (pH 3.5 and 0.3% bile (data not shown)), the best-performing strains were four *L. fermentum* (238, 508, SBS-1, and 1924), three *L. acidophilus* (LA11883, LX11873, and LX11850), *L. gasseri* LG11859, *L. salivarius* Ls-33, and *L. mucosae* LX11893. However, when the tolerance data under stronger conditions (pH 2.5 and 0.9% bile) were projected, eight of the best-performing strains were *L. acidophilus* (NCFM, LA11883, LA11890, LA11892, LA11897, 74-2, LA1076, and LX11873), and the remaining two were *L. fermentum* 508 and *L. reuteri* 1753 (Supplementary Materials Figure S9B). Thus, with regard to acid and bile tolerance, *L. acidophilus* strains were among the best-performing. In this comparison of the rank-ordered data, the top 10 strains at pH 2.5 and 0.9% bile did not show any significant differences in other parameters versus the rest of the strains, and among these strains, only 2 were commercial, i.e., *L. acidophilus* NCFM and 74-2 (Supplementary Materials Figure S9A).

In addition, the parameters were examined separately for the 13 strains isolated from the vaginal tract and compared with the rest of the strains in the rank-ordered data (Supplementary Materials Figure S10A). Tolerance to acid (*p* = 0.003 for pH 2.5 and *p* < 0.001 for pH 3.5) and bile (*p* = 0.004 for 0.3% and *p* = 0.002 for 0.9%) was lower in vaginally isolated strains versus the other strains in the panel. Furthermore, these strains produced more H_2_O_2_ and adhered better to VECs, albeit insignificantly.

When VEC adhesion was correlated to H_2_O_2_ production in the rank-ordered data in the full dataset (Supplementary Materials Figure S10B), the 10 best-performing strains were more diverse in origin, with 4 of vaginal origin (*L. crispatus* LX11797, *L. crispatus* LX11798, *L. jensenii* LX11794, and *L. jensenii* LX11796). The remainder of the 10 best-performing strains comprised *L. crispatus* LX1220, *L. jensenii* 911, *L. gasseri* 811, *L. johnsonii* LG0883, *L. fermentum* LX11865, and *L. reuteri* 1753. The top 10 strains showed good VEC adhesion and H_2_O_2_ production, but they had poor tolerance to mild acid (*p* = 0.042) and bile (*p* = 0.003 for 0.3% and *p* = 0.022 for 0.9%). Thus, if a strain shows good VEC adhesion and H_2_O_2_ production or is isolated from the vaginal tract, it does not necessarily have good tolerance to acid or bile.

### 3.7. Cluster Analysis of Rank-Ordered Data

**Figure 3:**
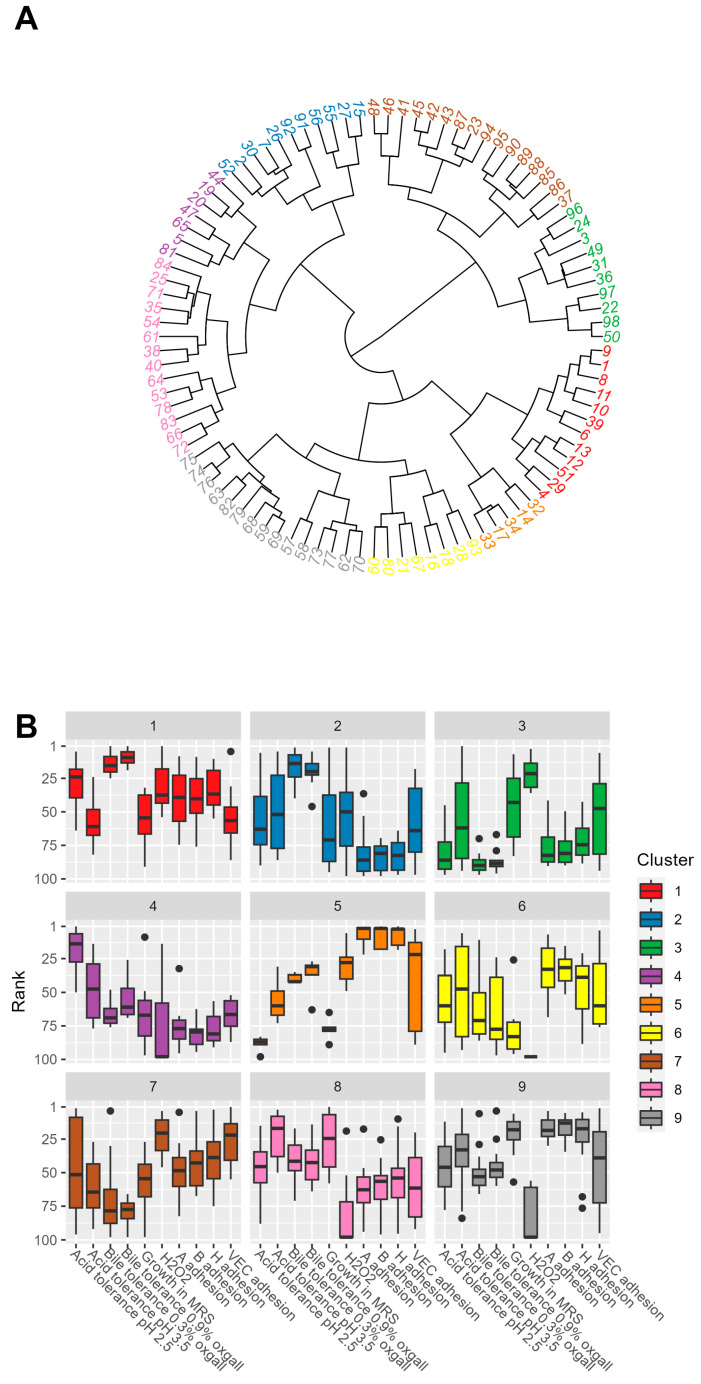
(**A**) Nine clusters formed from the rank-ordered data of the 98 strains using hierarchical clustering with Ward’s method. The numbers indicating the strains are depicted in Table 1. The quantity of bacteria in different clusters is as follows: cluster 1: 14; cluster 2: 12; cluster 3: 10; cluster 4: 16; cluster 5: 8; cluster 6: 14; cluster 7: 6; cluster 8: 7; and cluster 9: 11. (**B**) The biological parameters measured from the strains in each cluster represented by box plots. The box shows the interquartile range, the line inside the box indicates the median, whiskers indicate the minimum and the maximum values, and dots represent the outliers.

To better visualize the characteristics of the 98 strains, 9 clusters were created by hierarchical clustering according to Ward’s method (Figure 3A). Each cluster contained various *Lactobacillus* species, and none contained just one species. Cluster 8 had the most ‘classical’ probiotic characteristics (Figure 3B), with high tolerance to acid and bile and good growth in MRS. In terms of H_2_O_2_ production and adhesion to blood group antigens and VECs, the strains in cluster 8 were among the lowest-ranking. This cluster contained the most commercial strains (5 of 14), but otherwise, the commercial strains were distributed across seven clusters, with cluster 5 being the only one without commercial strains. Cluster 1 contained the second highest number of commercial strains (3 of 12), with good acid and bile tolerance but moderate to good rankings for other characteristics, apart from average adherence to VECs. This cluster contained most of the *L. acidophilus* strains (10 of 15 *L. acidophilus* strains in the panel).

With regard to the properties of vaginal probiotics, the most notable clusters were clusters 7, 5, and 9, showing high adhesion to VECs and/or high H_2_O_2_ production. Additionally, cluster 3 contained strains that produced significant H_2_O_2_ and adhered to VECs (Figure 3B). Cluster 5 (five strains) showed the best adherence to blood group antigens A, B, and H (Figure 3B). This cluster contained strains that were low in growth but tolerated bile and mild acidic conditions rather well and produced H_2_O_2_ moderately. Cluster 5 comprised *L. amylovorus* LX11898, *L. brevis* LX11864, and three *L. fermentum* (*L. fermentum* LX11852, *L. fermentum* LX11853, and *L. fermentum* LX11865). All of these strains were of fecal origin. The other cluster with good adherence to blood group antigens A, B, and H was cluster 9 (see below), which, likewise, did not contain any vaginal isolates. Strains from cluster 9 also adhered well to all blood group antigens, with good growth acid resistance and moderate bile resistance, but failed to produce H_2_O_2_. This cluster contained more than half of the *L. rhamnosus* strains (11 of 20 in the panel) and two *L. paracasei* and two *L. plantarum* strains; however, likewise, none was of vaginal origin.

Cluster 3, containing six different species (*L. acidophilus*, *L. fermentum*, *L. helveticus*, *L. delbrueckii bulgaricus*, *L. jensenii,* and *L. crispatus*) had the opposite profile to cluster 9, producing H_2_O_2_ well but adhering poorly to blood group antigens. Most of the *L. jensenii* strains (four of six strains) resided in this cluster. The other two *L. jensenii* strains were in cluster 7, with good H_2_O_2_ production and VEC adhesion, implying that the *L. jensenii* species has potential as a probiotic for vaginal health.

The vaginal isolates in the screening panel were distributed among clusters 2, 3, 6, and 7, indicating that lactobacilli from the vaginal tract have varying characteristics. Cluster 7, which was also the largest of the clusters, contained the most vaginal isolates (7 of 16 strains) (*L. crispatus* LX11211, *L. crispatus* LX12212, *L. crispatus* LX11797, *L. crispatus* LX11798, *L. crispatus* LX11799, *L. jensenii* 11794, and *L. jensenii* LX11795). These strains showed rather good adhesion to blood group antigens and VECs and good H_2_O_2_ production but moderate tolerance to acid and bile and growth in MRS.

## 4. Discussion

To elicit a health benefit, an oral probiotic strain should tolerate acid and bile, show good adhesion to intestinal or vaginal epithelia, depending on the target site, and possess antimicrobial properties. Furthermore, to be produced commercially, the strain should be culturable on a large scale. The aim of this study was to characterize the in vitro probiotic properties of 100 *Lactobacillus sensu lato* strains that were isolated primarily from the intestinal and vaginal tracts and rank them into various functional groups to determine their suitability for gastrointestinal and vaginal indications. Although important probiotic characteristics, such as H_2_O_2_ production, adhesion to vaginal epithelial cells, and bile and acid resistance were measured in this preliminary screening, other important probiotic safety-related properties, such as hemolysis and, most notably, antibiotic resistance [23], were not. Thus, further in-depth safety evaluation of the strains is needed for any future clinical studies, as has been done, for instance, by Pino and coworkers [24]. However, the results obtained in the current study can be utilized as a primary strain selection tool for specific health indications. The results show high strain-specificity in each characteristic tested, and the strains fell into nine variable clusters. The results confirm the widely held notion that probiotic properties are strain-specific [11]. None of the clusters was dominated by a single species. However, the clustering differentiated strains that tolerated bile and acid, indicating their potential for gastrointestinal indications. Furthermore, the clustering identified strains that adhered to vaginal cells and produced H_2_O_2_, conferring on them vaginal benefits.

Lactobacilli possess differential surface characteristics and express various enzymes, creating strain and species specificity in their response to environmental conditions and stresses [25]. Bile salts have strong antimicrobial potential, and tolerance to bile determines the ability to survive in the small intestine [26]. Resistance to bile parallels that to other stresses, such as acid and oxidative stress [25,26,27], which we also noted in our study. This tolerance differed based on the strength of such conditions, and our study included strains that varied in their tolerance. The ‘traditional’ probiotic species—*L. acidophilus* and *L. rhamnosus*— tolerated the stronger acid and bile conditions consistently, as expected, because the genetic machinery for bile and acid tolerance is well described for *Lactobacillus* [26]. All 15 *L. acidophilus* strains and most (16/20) *L. rhamnosus* strains were fecal in origin, and logically, the fecal isolates tolerated bile better than vaginal isolates in the rank-ordered data because the fecal strains had adapted to the conditions in the gastrointestinal tract.

Overall, the commercial strains were easier to culture, and 4 of the 10 easiest strains to culture were commercial, whereas none of 10 worst performers was. Ease of production, viability, and stability, are the key factors in the selection of commercial probiotics and are important for industrial-scale production. In addition, probiotics need to tolerate various stresses during production [3,28]. Thus, as expected, many of the easiest strains to culture were commercial strains. Conversely, *L. iners*, *L. brevis*, *L. amylovorus*, and *L. mucosae*, all of which are non-commercial strains, grew poorly in MRS. However, the small number of these strains in the panel might have biased this result; for instance, numerous *L. rhamnosus*, *L. crispatus*, and *L. fermentum* contained both strains that grew well and grew poorly.

Traditionally, adherence has been examined with intestinal epithelial cells, such as Caco-2, or mucus, such as that from pigs or humans [29]. In our study, we used a different approach, measuring the adherence of lactobacilli to histo-blood group antigens A, B, and H, as well as to VECs. The ABO histo-blood group system consists of two antigens (A and B) and four blood types (types A, B, AB, and O), of which group O expresses only H antigen, the biosynthetic precursor of antigens A and B. These antigens are widely expressed in red blood cells and many tissues and secretions, including the gastrointestinal and vaginal mucosae [30]. However, ABH antigens are not present in all individuals; non-secretors lack functional fucosyltransferease-2 and do not express these antigens in their secretions or mucosa, instead expressing Lewis A antigen [31].

Some pathogens and their toxins can bind blood group antigens directly [32], such as *H. pylori* [33], Norwalk virus [34], norovirus GII.4 genotype [35], and rotavirus [36]. Lactobacilli strains of *L. crispatus*, *L. mucosae*, *L. plantarum,* and *L. paracasei* express blood group antigen-binding adhesins and therefore may compete in adhesion mechanisms that impede pathogenesis [22,37,38,39,40]. Moreover, blood group antigens, glycans on ABO antigens, and Lewis antigen shed into the intestinal lumen can be fermented by intestinal bacteria such as bifidobacteria and *Bacteroides* spp.; thus, these glycans can serve as energy sources for bacteria and affect the composition of the gut microbiota [14]. Research shows that the composition of intestinal mucosal microbiota is affected by the ABO blood type and the secretor status of the host [14,41], especially the presence of B antigen [39], although contradictory results have also been reported [42]. However, information on the adhesion properties of probiotics to different blood group antigens could be relevant in the design of personalized probiotics for the market because specific adhesins toward a certain blood group antigen could further increase the colonization potential of probiotics. In our study, strains were differentiated and ranked as having antigen-specific adhesion, non-specific adhesion (similar affinity to all), or equal affinity (highest median adhesion). This categorization could be used to tailor probiotics based on adherence toward a certain blood group antigen or implement a more generic approach that is suitable for most consumers. Among the top 10 strains with the most variability in their adhesion to blood group antigens, 2 were commercial (*L. rhamnosus* HN001 and *L. rhamnosus* Lr-32).

It was previously reported that bacterial survival in an upper gastrointestinal experimental model depended on the secretor and non-secretor status of the donor, with bacteria from the latter being more vulnerable to acid and bile [43]. However, we do not know whether the donor from which the fecal strains were isolated was a non-secretor or secretor; thus, ideally, future studies should harvest bacteria from individuals with different blood group antigens and secretor statuses. Notably, fecal strains adhered better to all blood group antigens compared with strains sourced elsewhere. Furthermore, clustering of the rank-ordered data showed that adhesion to antigens A and B by some strains was associated with poor acid and bile tolerance, indicating that they would survive poorly in the upper gastrointestinal tract, although there were still some clusters that had both good acid and bile tolerance and good adhesion to blood group antigens. However, identifying new probiotic candidates solely from among those that best tolerate acid and bile would overlook other beneficial properties. For instance, the production of H_2_O_2_, an asset of vaginal lactobacilli, correlated negatively with tolerance to acid and bile. Considering women’s health, such tolerance might be dispensable for intravaginal probiotics, whereas the bacteria in oral supplements would first need to travel through the gastrointestinal tract, resisting the acidity of the stomach and the bile from liver [26]. To this end, encapsulation technologies can be used to overcome such poor survival [44].

Vaginal bacteria can be classified into five main types by community. Four are predominated by *Lactobacillus* spp.—*L. crispatus*, *L. gasseri*, *L. iners*, and *L. jensenii*—whereas the fifth comprises a mixed community of strictly anaerobic bacteria [45]. Lactobacilli protect the vaginal mucosa through adherence to the vaginal epithelia and their antimicrobial properties [46]. The production of lactic acid, H_2_O_2_, and antimicrobial agents is considered a beneficial attribute of vaginal lactobacilli [9]. Whereas lactic acid inhibits the growth of pathogenic bacteria by lowering pH, the function of H_2_O_2_ is not fully elucidated, but it has been suggested to destroy bacteria owing to a lack of H_2_O_2_-degrading enzymes such as catalase peroxidase [9,47]. Nevertheless, 70% to 95% of lactobacilli in the vaginal microbiota of healthy women produce H_2_O_2_, and the levels of these lactobacilli decrease in women with vaginal infections [48,49,50]. *L. gasseri*, *L. jensenii,* and *L. crispatus* species commonly predominate in the vaginal tracts of healthy women [10], and more than 90% of the strains in these genera produce H_2_O_2_ [48]. For instance, at least 80% of isolates of *L. jensenii*, *L. salivarius*, *L. rhamnosus*, and *L. vaginalis* generate high amounts of H_2_O_2_, as does *L. gasseri*, although to a lesser extent [51]. In our study, 53% (52/98) of strains produced H_2_O_2_, the most prominent of which were *L. gasseri*, *L. jensenii,* and *L. crispatus*, whereas *L. rhamnosus*, *L. johnsonii,* and *L. brevis* synthesized lower amounts. However, most strains in the screening panel were of fecal rather than vaginal origin. Most of the vaginal isolates (10/13) produced H_2_O_2_, with the exception of *L. iners*. This was expected because *L. iners* strains are reported to generate limited amounts of H_2_O_2_ [52]. Furthermore, *L. iners* is not well adapted to grow in MRS, preferring sheep blood agar. *L. iners* also produce L-lactic acid but not D-lactic acid, which is regarded as a more protective isomer for vaginal health [52]. The function of *L. iners* in vaginal health is controversial; it is commonly found in healthy women but is also often encountered during vaginal dysbiosis [52,53,54]. Moreover, the *L. iners* strains adhered poorly to VECs in our study compared with the *L. crispatus* and *L. jensenii* strains. Notably, *L. iners* lack adhesins that are common to other lactobacilli, instead expressing fibronectin-binding protein, which is similar to pathogenic *Staphylococcus aureus* [52].

In dysbiosis of the vaginal microbiota, such as during BV, lactobacilli are depleted from the vaginal microbiota and are overrun by various facultative anaerobes [55,56]. Probiotic lactobacilli with or without antibiotics administered orally or intravaginally have shown promise in reducing the risk of vaginal infections [55,57,58]. Good adherence of lactobacilli to vaginal epithelia is a potential mechanism for protecting the vaginal mucosa from pathogenesis by inhibiting and interfering with the adhesion of pathogenic microorganisms [46]. In our study, the adhesion of lactobacilli to VECs correlated positively with good H_2_O_2_ production. In the rank-ordered data, when the entire dataset was projected according to VEC adhesion and H_2_O_2_ production, 4 of the top 10 strains were vaginal in origin, highlighting their potential as probiotics for vaginal health. These vaginal isolates, especially *L. jensenii*, also showed lower tolerance to acid and bile compared with the rest of the strains. Future studies should compare the responses of less tolerant species, such as *L. jensenii,* to those with greater tolerance, such as *L. acidophilus* and *L. plantarum*, and determine whether gradual exposure to increasing stress improves tolerance in *L. jensenii* [26].

One drawback of our study is the use of bovine bile (Oxgall), which slightly reduces the pH of culture media [59]. We did not control the pH of the culture medium in the bile tolerance assays, as has been done in some other studies [60]; thus, the lower viability in bile could have been caused by the accumulation of lactic acid and other organic acids that are produced by the bacteria [26]. To survive passage through the gastrointestinal tract, the adaptation of microorganisms to a stressor might enhance survival under another stressor that is encountered [61]. This species-specific cross protection can be beneficial when cells are exposed to a combination of stresses [61], and these adaptive responses can be addressed further in examining the probiotic properties of lactobacilli.

In conclusion, we screened 98 strains for their overall growth in MRS, tolerance to acid and bile, H_2_O_2_ production, and adhesion to VECs and blood group antigens. There were clear differences in these characteristics depending on the source of the strain, but there were also strain-dependent properties between species. We found that strains that were of fecal origin adapted better to acidic and high-bile conditions, whereas vaginal strains, with the exception of *L. iners*, were good H_2_O_2_ producers and adhered well to VECs, although some of these strains were less tolerant to acid and bile. Future encapsulation technologies and an improved understanding of the genetic background in the development of tolerance and beneficial properties can guide and improve the selection of probiotics.

## Figures and Tables

**Table 1:** List of lactobacilli strains that were screened in the study, as well as their commercial category and source. For strains marked with an asterisk (*), the taxonomic classification to a species was not absolutely certain. Strains marked with a hashtag (#) did not grow under laboratory conditions and were excluded from future studies. The category in the table indicates whether the strain is commercial (Com.) or non-commercial (Non-Com.). Source in the table refers to origin of the strain as fecal (F), unknown (U), dairy (D), plant (P), or vaginal (V). The color coding and number for the various parameters measured in this study indicate the rank order of the strains in the measured parameters, with 1 (red) indicating the best-performing strain and 98 (blue) indicating the worst-performing strain.

Num	Taxonomic Species	Strain	Category	Source	Bile Tolerance 0.3% Oxgall	Bile Tolerance 0.9% Oxgall	Acid Tolerance pH 2.5	Acid Tolerance pH 3.5	Growth in MRS	A Adhe-sion	B Adhe-sion	H Adhe-sion	VEC Adhe-sion	H_2_O_2_
1	*Lactobacillus acidophilus*	NCFM	Com.	F	16	6	21	49	39	46	48	44	81	37
2	*Lactobacillus acidophilus*	LA11871	Non-com.	F	14	8	47	43	71	36	91	94	64	42
3	Lactobacillus acidophilus *	LA0893	Non-com.	F	97	89	91	11	16	42	63	42	83	34
4	*Lactobacillus acidophilus*	LA11883	Non-com.	F	15	13	25	24	32	23	9	15	56	43
5	*Lactobacillus acidophilus*	LA11890	Non-com.	F	57	26	1	77	67	96	94	91		55
6	*Lactobacillus acidophilus*	LA11892	Non-com.	F	18	19	13	63	48	74	76	33	42	38
7	*Lactobacillus acidophilus*	LA11897	Non-com.	F	22	20	27		46	86	81	82	72	47
8	*Lactobacillus acidophilus*	74-2	Com.	F	8	2	19	82	66	43	43	41	86	39
9	*Lactobacillus acidophilus*	LA1076	Non-com.	F	20	9	16	59	38	58	58	46	68	48
10	*Lactobacillus acidophilus*	LA11884	Non-com.	F	24	15	32	66	35	19	38	52	65	12
11	*Lactobacillus acidophilus*	La-14	Com.	F	21	12	44	64	36	56	62	55	51	5
12	*Lactobacillus acidophilus*	LA11880	Non-com.	F	25	16	39	78	61	8	15	10	48	20
13	*Lactobacillus acidophilus*	LA11872	Non-com.	F	12	10	41	72	68	22	26	12	63	45
14	*Lactobacillus amylovorus*	LX11898	Non-com.	F	37	63	98		89	22	18	15	79	6
15	*Lactobacillus amylovorus*	LX11891	Non-com.	F	26	18	90	51	29	93	93	93	47	25
16	*Levilactobacillus brevis*	Lbr-35	Com.	U	11	24	69	56	70	64	30	54	16	98
17	*Levilactobacillus brevis*	LX11864	Non-com.	F	35	27	83	65	65	10	18	18	3	49
18	*Levilactobacillus brevis*	LX11860	Non-com.	F	33	38	95	81	85	30	28	31	4	98
19	*Lacticaseibacillus casei*	Lc-11	Com.	D	76	68	29	48	9	89	80	82	78	98
20	*Lacticaseibacillus paracasei*	LC11896	Non-com.	F	67	54	50		49	80	77	72	87	98
21	*Lacticaseibacillus rhamnosus*	LC11868	Non-com.	F	80	97	72	39	73	18	34	29	75	98
22	*Lactobacillus crispatus*	LX1307	Non-com.	U	81	87	93	75	22	90	89	86	32	7
23	*Lactobacillus crispatus*	LX1220	Non-com.	U	87	76	54	71	43	61	68	62	12	8
24	*Lactobacillus crispatus*	LX1308	Non-com.	U	85	96	71	67	41	48	50	61	93	32
25	*Limosilactobacillus fermentum*	238	Non-com.	U	27	59	48	9	15	86	86	84	80	98
26	*Limosilactobacillus fermentum*	508	Non-com.	U	3	21	6	12	94	77	70	82	88	51
27	*Lactobacillus crispatus*	LX0152	Non-com.	U	40	46	82	53	21	88	75	74	36	29
28	*Latilactobacillus curvatus*	360	Non-com.	U	86	82	51	93	93	68	52	42	33	98
29	*Lactobacillus acidophilus*	LX11873	Non-com.	F	7	3	23	25	72	30	22	20	31	54
30	*Lactobacillus acidophilus*	LX11850	Non-com.	F	19	11	63	16	84	96	94	92	70	50
31	*Lactobacillus delbrueckii* spp.*bulgaricus*	LB0064	Com.	D	91	90	45	30	34	82	82	57	77	31
32	*Limosilactobacillus fermentum*	LX11852	Non-com.	F	42	30	89	55	79	2	2	3	89	28
33	*Limosilactobacillus fermentum*	LX11853	Non-com.	F	42	31	85	31	78	2	1	1	22	40
34	*Limosilactobacillus fermentum*	LX11865	Non-com.	F	43	37	87	73	76	2	2	3	13	24
35	*Limosilactobacillus fermentum*	SBS-1	Com.	U	28	33	58	8	52	73	39	70	90	98
36	*Limosilactobacillus fermentum*	LX1312	Non-com.	U	70	67	55	1	7	68	72	66	45	13
37	*Limosilactobacillus fermentum*	LX11866	Non-com.	F	82	83	84	70	86	5	4	3	43	15
38	*Limosilactobacillus fermentum*	2342	Non-com.	U	39	34	15	7	58	53	57	50	38	98
39	*Limosilactobacillus reuteri*	1753	Non-com.	U	9	7	5	46	62	59	48	40	5	27
40	*Limosilactobacillus fermentum*	1924	Non-com.	U	17	32	56	3	42	79	84	75	37	98
41	*Lactobacillus gasseri*	LG11859	Non-com.	F	4	70	7	27	87	82	26	24	46	18
42	*Lactobacillus gasseri*	811	Non-com.	U	83	66	9	61	30	67	66	60	23	9
43	*Lactobacillus gasseri*	LG0179	Non-com.	U	64	71	11	44	60	47	46	38	11	33
44	*Lactobacillus gasseri*	Lg-36	Com.	F	72	69	3	14	63	77	62	56	66	98
45	*Lactobacillus johnsonii*	LG0883	Non-com.	U	79	78	4	40	44	66	68	72	21	11
46	*Lactobacillus gasseri*	LG11895	Non-com.	F	30	80	8	68	53	38	37	44	55	30
47	*Lactobacillus gasseri*	LG11876	Non-com.	F	69	65	10	23	97	68	78	81	67	14
48	*Lactobacillus johnsonii*	LG0884	Non-com.	U	32	77	2	74	27	28	24	21	35	17
49	*Lactobacillus helveticus*	LH0138	Com.	D	88	86	77	28	45	90	90	88	28	21
50	*Lactobacillus jensenii*	911	Non-com.	U	89	79	81	90	82	87	86	72	7	3
51	*Limosilactobacillus mucosae*	LX11854	Non-com.	F	1	1	64	58	91	36	34	28	57	1
52	*Limosilactobacillus mucosae*	LX11893	Non-com.	F	13	5	30	5	74	53	78	72	27	2
53	*Lacticaseibacillus paracasei*	Lpc-37	Com.	D	71	62	34	45	25	60	54	53	41	98
54	*Lactiplantibacillus plantarum*	Lp-115	Com.	P	23	14	53	36	5	70	72	59	92	98
55	*Lactiplantibacillus plantarum*	LX11855	Non-com.	F	31	28	79	69	69	97	97	96	18	98
56	*Lactiplantibacillus plantarum*	LX11878	Non-com.	F	10	17	66	86	2	98	98	97	29	98
57	*Lactiplantibacillus plantarum*	LX11861	Non-com.	F	29	25	60	79	10	9	6	5	74	98
58	*Lactiplantibacillus plantarum*	LX11856	Non-com.	F	6	4	65	84	37	26	28	18	71	98
59	*Lacticaseibacillus rhamnosus*	LX11869	Non-com.	F	53	49	46	37	33	10	8	10	54	56
60	*Lacticaseibacillus rhamnosus*	LX11870	Non-com.	F	56	81	73	10	96	7	46	87	61	98
61	*Lacticaseibacillus paracasei*	LX11886	Non-com.	F	36	35	31	50	47	94	26	33	69	98
62	*Lacticaseibacillus paracasei*	LX11858	Non-com.	F	62	50	74	34	8	4	12	76	82	98
63	*Lacticaseibacillus rhamnosus*	LX11875	Non-com.	F	66	60	61	21	23	29	22	28	9	62
64	*Lacticaseibacillus rhamnosus*	HN001	Com.	D	44	56	20	17	4	18	58	64	34	57
65	*Lacticaseibacillus rhamnosus*	LX11888	Non-com.	F	74	61	14	47	88	74	86	64	52	61
66	*Lacticaseibacillus rhamnosus*	LX11867	Non-com.	F	49	44	37	38	31	54	52	10	60	63
67	*Lacticaseibacillus rhamnosus*	LX11882	Non-com.	F	84	74	24	6	26	15	18	21	73	98
68	*Lacticaseibacillus rhamnosus*	LX11863	Non-com.	F	59	55	40	62	18	18	10	18	26	58
69	*Lacticaseibacillus rhamnosus*	LX11851	Non-com.	F	55	57	52	22	20	22	22	14	39	60
70	*Lacticaseibacillus rhamnosus*	Lr-32	Com.	U	38	47	22	32	11	21	12	68	95	98
71	*Lacticaseibacillus paracasei*	LX11885	Non-com.	F	47	41	36	4	12	50	55	96	85	98
72	*Lacticaseibacillus rhamnosus*	LX11874	Non-com.	F	61	51	43	19	24	66	96	16	63	52
73	*Lacticaseibacillus rhamnosus*	LX11877	Non-com.	F	50	45	33	20	28	18	13	36	58	98
74	*Lacticaseibacillus rhamnosus*	LX11879	Non-com.	F	52	42	28	54	13	22	34	17	25	98
75	*Lacticaseibacillus paracasei*	LX11887	Non-com.	F	63	52	12	26	57	12	22		2	98
76	*Lacticaseibacillus rhamnosus*	LX11862	Non-com.	F	51	48	35	33	19	24	28	24	24	98
77	*Lacticaseibacillus rhamnosus*	LX11857	Non-com.	F	45	36	59	35	17	30	15	12	91	98
78	*Lacticaseibacillus rhamnosus*	1704	Non-com.	U	46	53	68	41	3	66	65	55	20	98
79	*Lacticaseibacillus rhamnosus*	LX11881	Non-com.	F	54	43	17	2	14	8	6	8	8	59
80	*Lacticaseibacillus paracasei*	LX11849	Non-com.	F	77	94	18	18	81	36	16	88	59	98
81	*Lacticaseibacillus rhamnosus*	LX11889	Non-com.	F	48	40	26	76	77	32	92	90	53	98
82	*Lacticaseibacillus rhamnosus*	LR1049	Non-com.	U	60	58	78	13	6	16	12	10	15	98
83	*Ligilactobacillus ruminis*	LX11894	Non-com.	F	68	29	76		56	46	48	46	44	19
84	*Ligilactobacillus salivarius*	Ls-33	Com.	U	34	64	88	15	1	58	56	48	84	98
85	*Lactococcus lactis*	LI-23	Com.	D	93	85	80	60	55	45	53	34	40	4
86	*Lactobacillus crispatus*	LX11211	Non-com.	V	78	75	57	92	98	32	29	24	49	44
87	*Lactobacillus crispatus*	LX11797	Non-com.	V	75	72	49	52	64	52	58	75	10	23
88	*Lactobacillus crispatus*	LX12212	Non-com.	V	73	84	75	42	54	60	42	40	30	46
89	*Lactobacillus crispatus*	LX11798	Non-com.	V	90	95	67	29	80	39	36	28	17	26
90	*Lactobacillus crispatus*	LX11799	Non-com.	V	58	73	86	83	51	43	44	36	1	35
91	*Lactobacillus iners#*	Li25-34	Non-com.	V										
92	*Lactobacillus iners*	Li19-22	Non-com.	V	2	23	62	85	95	76	72	66	97	53
93	*Lactobacillus iners*	Li21-23	Non-com.	V	5	22	70	80	90	78	76	64	96	98
94	*Lactobacillus iners#*	Li22-26	Non-com.	V										
95	*Lactobacillus iners*	Li14-7	Non-com.	V	65	39	42	89	92	40	40	36	76	98
96	*Lactobacillus jensenii*	LX11794	Non-com.	V	96	92	38	91	40	60	42	51	19	10
97	*Lactobacillus jensenii*	LX11795	Non-com.	V	98	98	96	87	59	50	64	52	14	41
98	*Lactobacillus jensenii*	LX12216	Non-com.	V	92	91	92	57	75	72	73	78	94	16
99	*Lactobacillus jensenii*	LX12217	Non-com.	V	95	93	97	94	50	84	80	84	50	36
100	*Lactobacillus jensenii*	LX11796	Non-com.	V	94	88	94	88	83	88	88	78	6	22

## Data Availability

Not applicable.

## Supplementary Materials

The following supporting information can be downloaded at: https://www.mdpi.com/article/10.3390/microorganisms11020329/s1, Figure S1: Growth on MRS at the strain level. Figure S2: Acid tolerance at the strain level. Figure S3: Bile tolerance at the strain level. Figure S4: Hydrogen peroxide production at the strain level at 3 hours. Figure S5: Adhesion to vaginal epithelial cells at the strain level. Figure S6: Adhesion to blood group antigens at the strain level. Figure S7: Box plots of measured characteristics of the lactobacilli isolated from feces in comparison to strains of non-fecal origin. Figure S8: Characteristics of the 16 studied commercial probiotics in comparison to non-commercial strains. Figure S9: Characteristics of the 10 best-performing strains with rank-ordered acid (pH 2.5) and strong bile (0.9% oxgall) tolerances in comparison to the other strains in the dataset. Figure S10: Characteristics of the 13 vaginally isolated lactobacilli in comparison to strains from other origins. Table S1: Statistical analysis of growth in MRS OD600. Table S2: Statistical analysis of acid tolerance at pH 3.5 (log reduction). Table S3: Statistical analysis of acid tolerance at pH 2.5 (log reduction). Table S4: Statistical analysis of bile tolerance, growth in 0.3% oxgall, % of growth in MRS w/o bile. Table S5: Statistical analysis of bile tolerance, growth in 0.9% oxgall % of growth in MRS w/o bile. Table S6: Statistical analysis of hydrogen peroxide production at 3 h% of comparator strain. Table S7: Statistical analysis of epithelial cell adhesion % of adhesion of comparator. Table S8: Statistical analysis of average A antigen adhesion % of adhesion of comparator. Table S9: Statistical analysis of average B antigen adhesion % of adhesion of comparator. Table S10: Statistical analysis of average H antigen adhesion % of adhesion of comparator.
