# Supplementary material for: In Vitro Screen of Lactobacilli Strains for Gastrointestinal and Vaginal Benefits
Source: Microorganisms. 2023 Jan 28;11(2):329. doi: 10.3390/microorganisms11020329 (PMC9967617; doi:10.3390/microorganisms11020329)
Supplement: Supplementary file 1 [file microorganisms-11-00329-s001.zip › microorganisms-2158555-supplementary.pdf]

Supplementary Figures

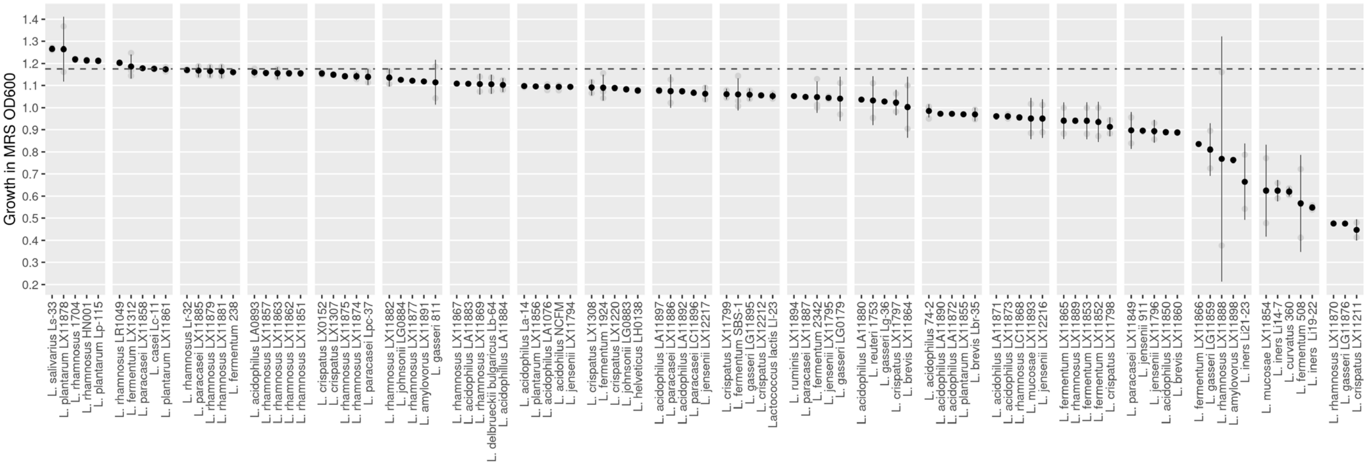

Supplementary figure 1. Growth on MRS at strain level. Two *L. iners* strains from the panel did not grow and were omitted from the figure. The data is expressed as mean ± SD absorbance at OD_{600}. The dashed line indicates the absorbance at OD_{600} obtained from the comparator strain *L. rhamnosus* GG.

2A)

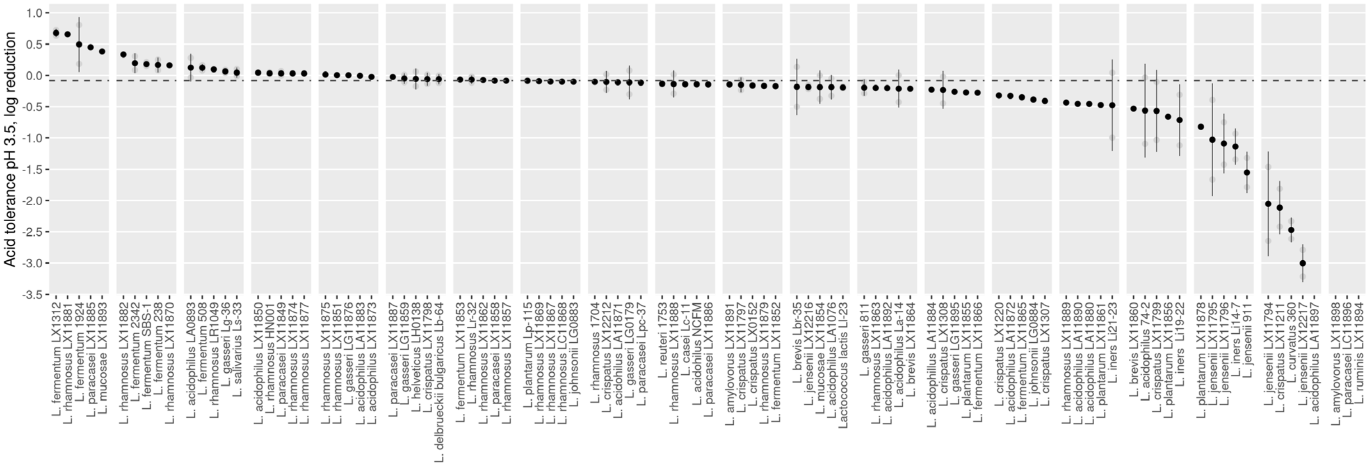

2B)

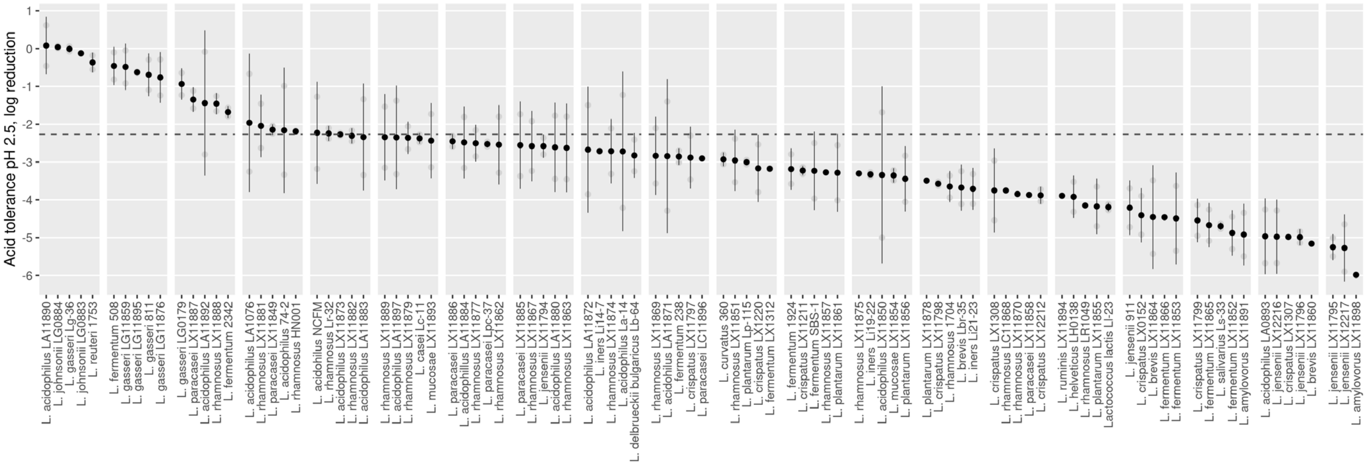

Supplementary figure 2. Acid tolerance at strain level. A) acid tolerance in pH 3.5. B) Acid tolerance in pH 2.5. Data was missing at pH 3.5 for *L. acidophilus* LA11897, *L. amylovorus* LX11898, *L. paracasei* LC11896, and *L. ruminis* LX11894. Two *L. iners* strains from the panel did not grow and were omitted from the figure. The data is expressed as a mean ±SD log reduction in comparison to value obtained at pH 7.2. The dashed line indicates the value obtained from the comparator strain *L. rhamnosus* GG.

3A)

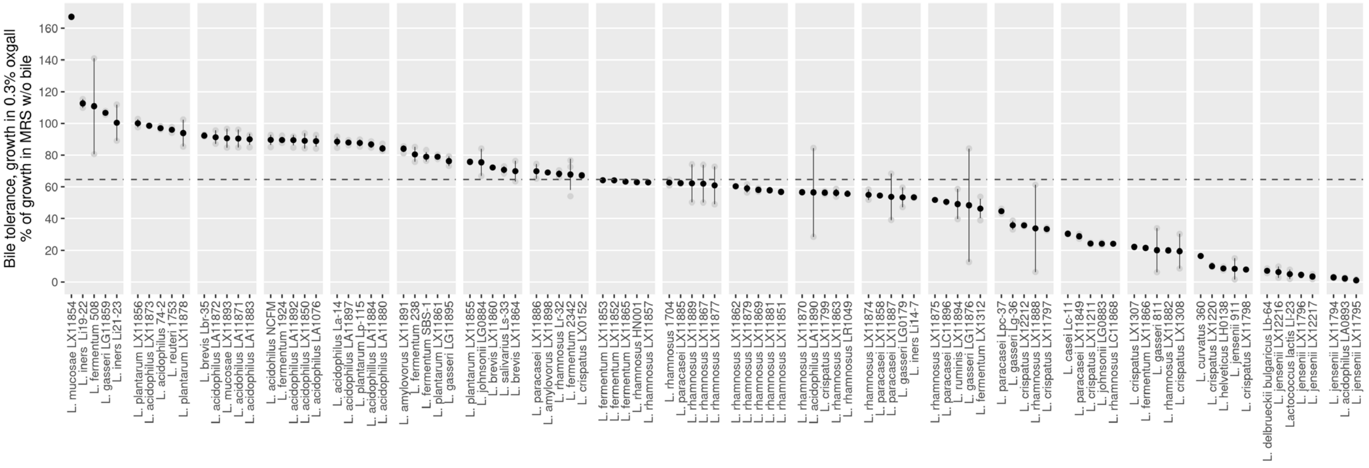

3B)

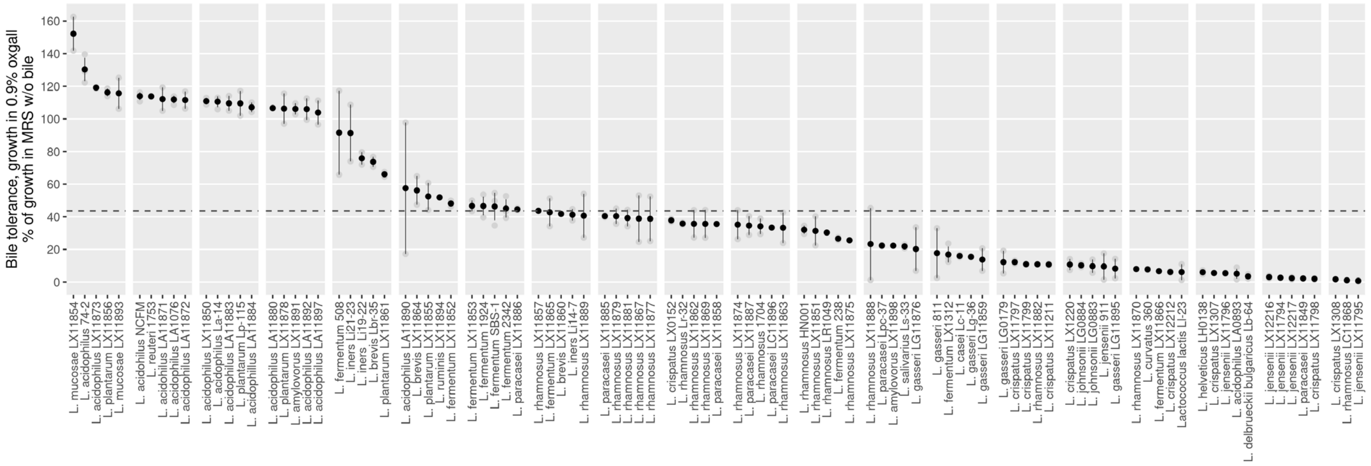

Supplementary figure 3. Bile tolerance at strain level. A) Bile tolerance in 0.3 % Oxgall and B) Bile tolerance in 0.9 % Oxgall. The data is expressed as mean ± SD percentage from the growth observed in culture medium without Oxgall. Two *L. iners* strains from the panel did not grow and were omitted from the figure. The dashed line indicates the value obtained from the comparator strain *L. rhamnosus* GG.

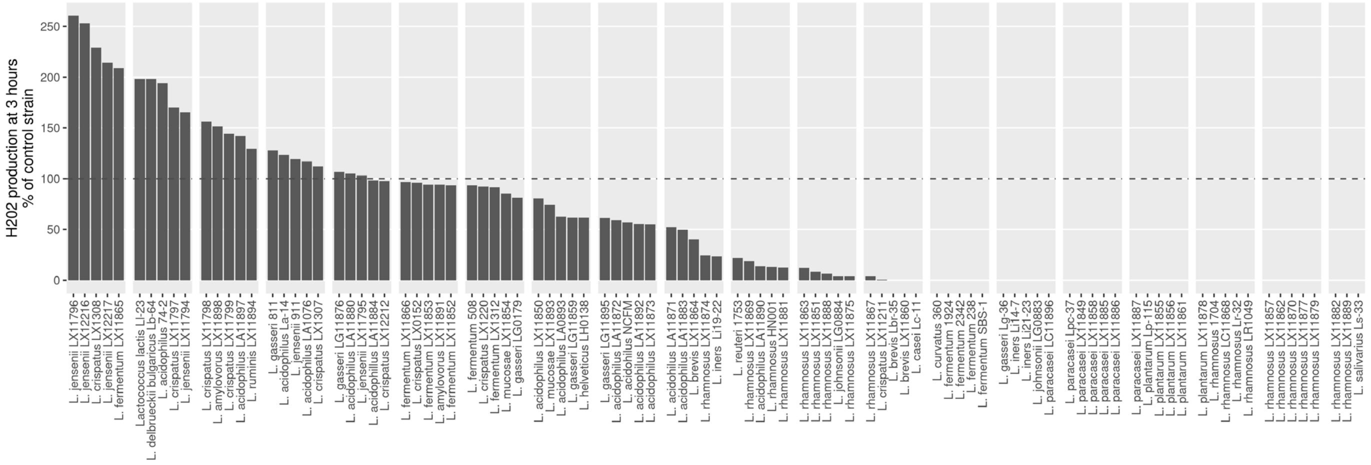

Supplementary figure 4. Hydrogen peroxide production at strain level at 3 hours. The production of hydrogen peroxide was calculated in relation to comparator strain *L. jensenii* DSM 20557 at 3 h time point. *L. brevis* Lbr-35, *L. brevis* LX11860, *L. casei* Lc-11, *L. curvatus* 360, *L. fermentum* SBS-1, *L. fermentum 238, L. fermentum* 2342, *L. fermentum* 1924, *L. gasseri* Lg-36, *L. iners* Li21-23, *L. iners* Li14-7, *L. johnsonii* LG0883, *L. rhamnosus* LX11870, *L. rhamnosus* LC11868, *L. rhamnosus* LX11882, *L. rhamnosus* Lr-32, *L. rhamnosus* LX11877, *L. rhamnosus* LX11879, *L. rhamnosus* LX11862, *L. rhamnosus* LX11857, *L. rhamnosus* 1704, *L. rhamnosus* LX11889, *L. rhamnosus* LR1049, *L. salivarius* Ls-33, all *L. paracasei* and all *L. plantarum* strains did not produce H_2O_2 at all. The dashed line indicates the value obtained from the comparator strain *L. jensenii* DSM 20557.

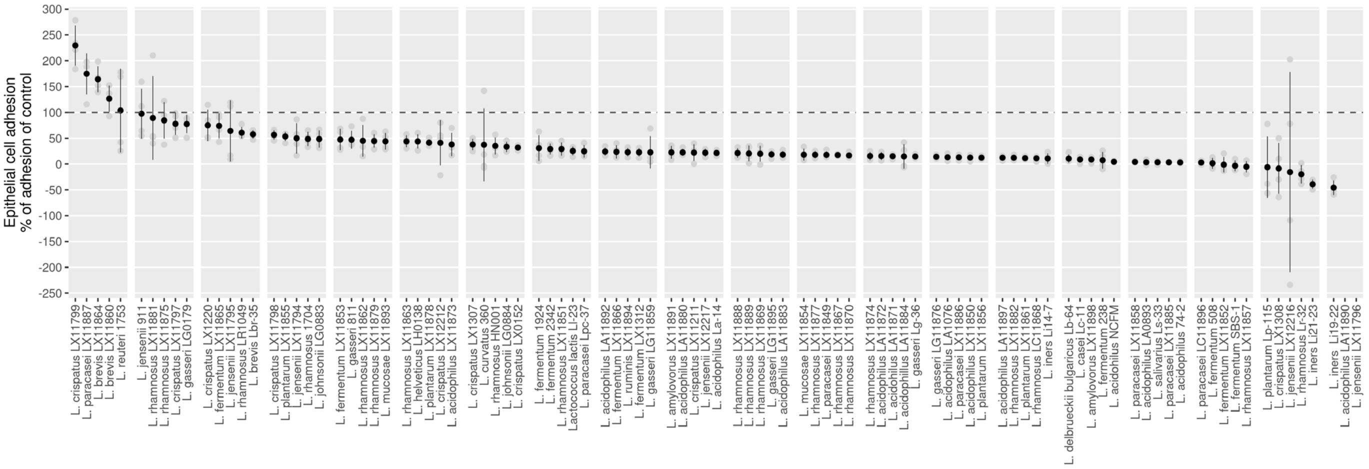

Supplementary figure 5. Adhesion to vaginal epithelial cells at strain level. Two *L. iners* strains from the panel did not grow and were omitted from the figure. The results are expressed as epithelial cell adhesion % of adhesion of *L. jensenii* LX11796. Mean± SD is shown for each strain. The dashed line represents the comparator strain *L. jensenii* LX11796.

6A)

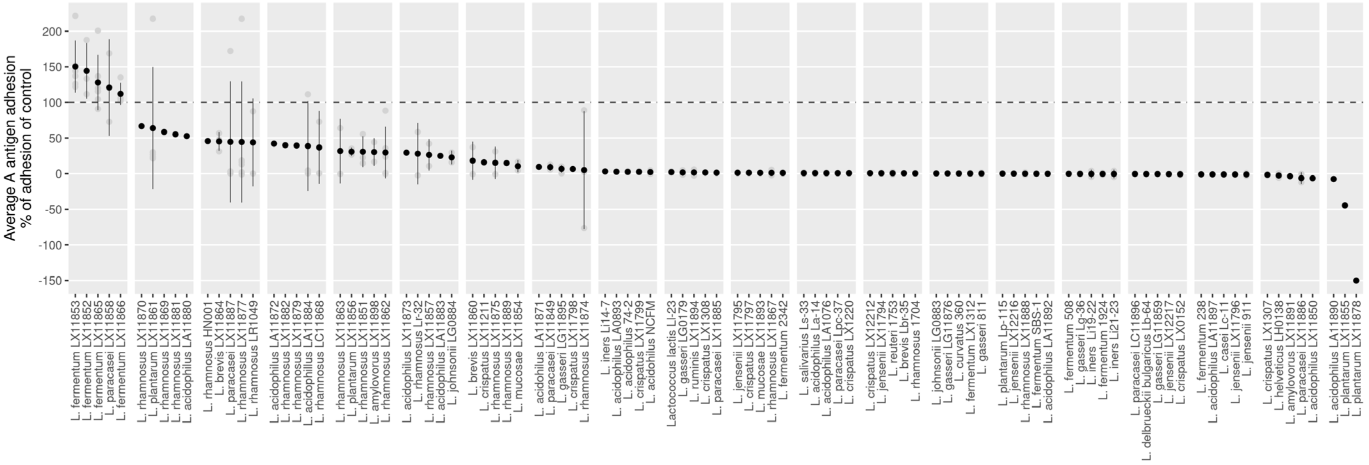

6B)

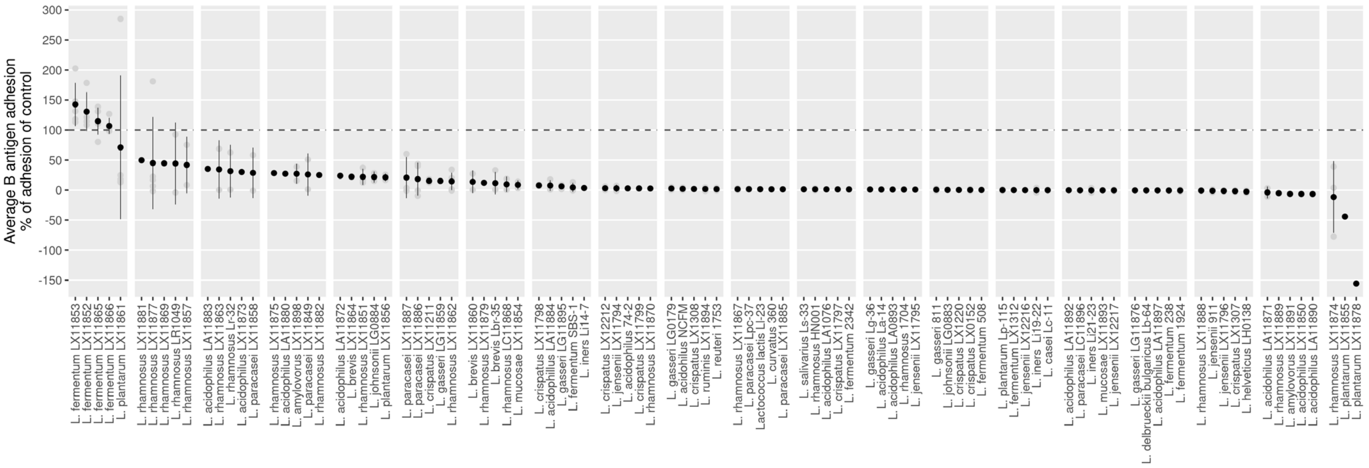

6C)

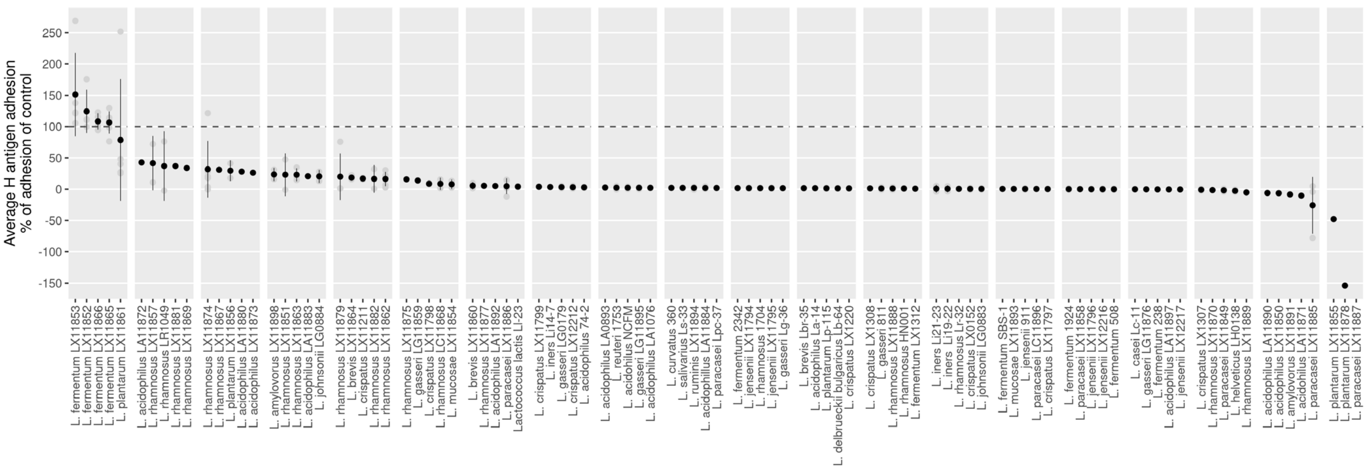

Supplementary figure 6. Adhesion to blood group antigen at strain level. A) Adhesion to blood group antigen A, B) Adhesion to blood group antigen B, and C) Adhesion to blood group antigen H at strain level. The dashed line is the adhesion of control strain *L. crispatus* LMG18199, that is known to adhere well to the A blood group antigen. The result for one strain, *L. paracasei* LX11887 is missing for the H antigen adhesion. The results are expressed as average antigen adhesion % of adhesion of *L. crispatus* LMG 18204, mean ± SD is shown for each strain.

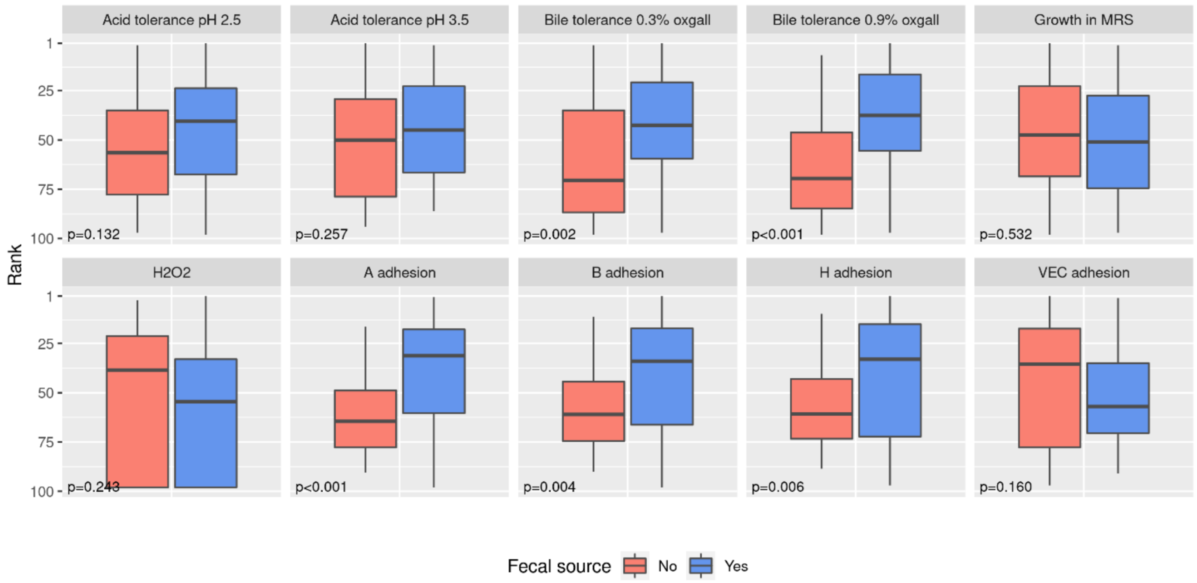

Supplementary figure 7. Box plots of measured characteristics of the lactobacilli isolated from feces (blue, rank -ordered data) in comparison to strains from non-fecal origin (red, rank-ordered data). The box shows the interquartile range, the line inside the box indicates the median, whiskers the minimum and the maximum values, and dots represent the outliers

8A)

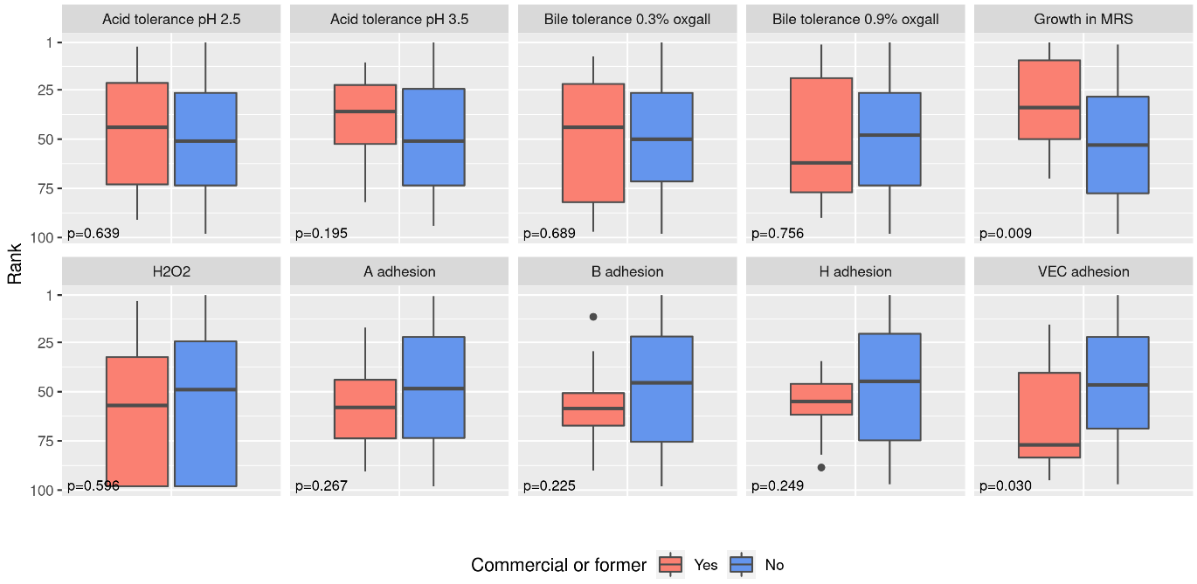

8B)

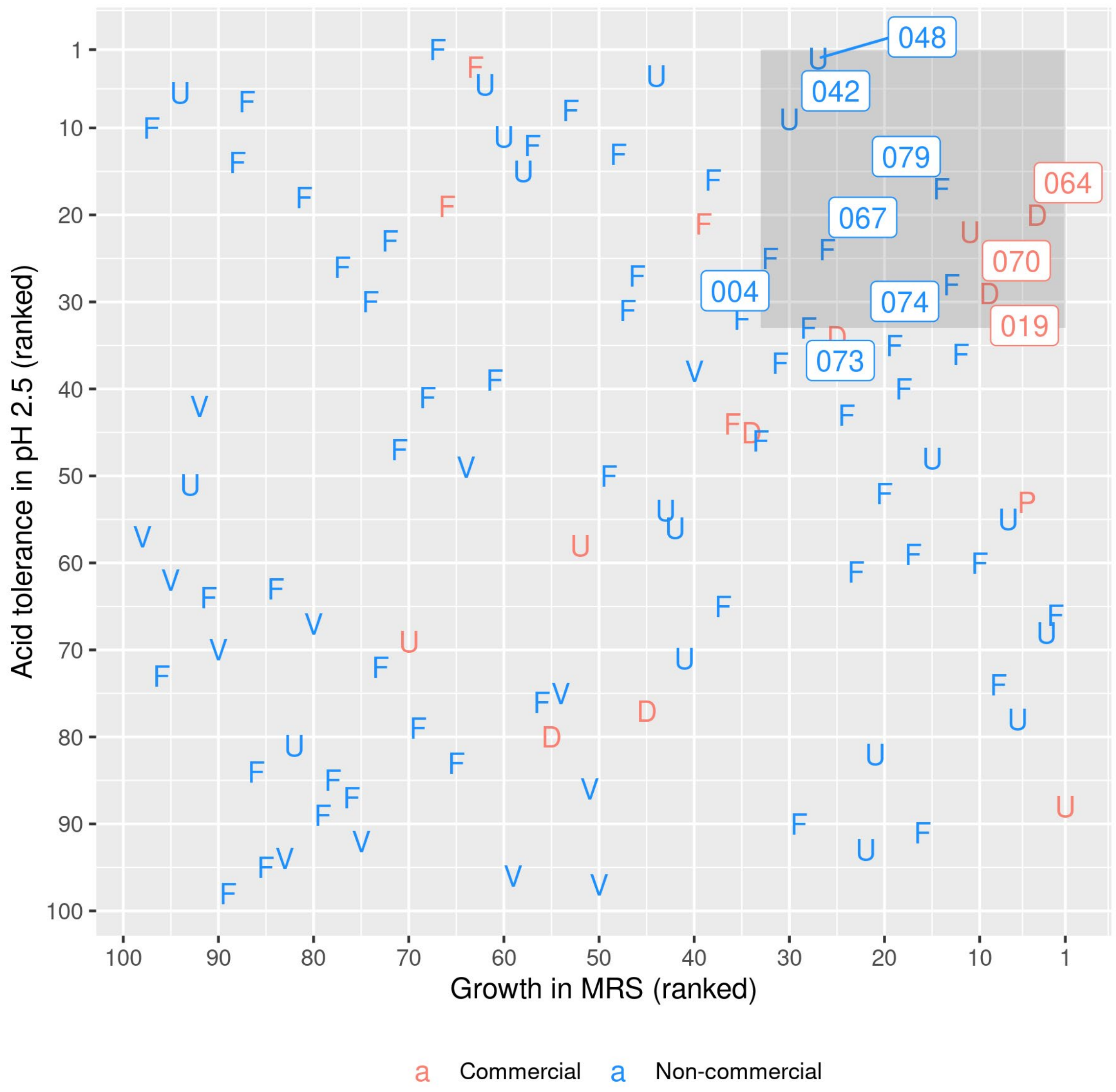

Supplementary figure 8. A) Characteristics of the studied 16 commercial probiotics (red rank-ordered data) in comparison to non-commercial strains (blue, rank-ordered data). In the analysis of rank ordered data of commercial strains was compared to non-commercial strains with respect of different parameters analyzed in the study. The box shows the interquartile range, the line inside the box indicates the median, whiskers the minimum and the maximum values, and dots represent the outliers. B) Data projection of rank-ordered data (strong acid tolerance (pH 2.5) versus growth in MRS) in two dimensions. In the top right corner 10 strains that perform the best in terms of acid tolerance at pH 2.5 and growth in MRS are indicated. Different letters indicate the different sources of strains F= Fecal, D = dairy, P= plant, V = vagina, U = unknown and colors whether the strain is commercial (red) or non-commercial (blue). The numbers indicating the strains are depicted on Table 1.

9A)

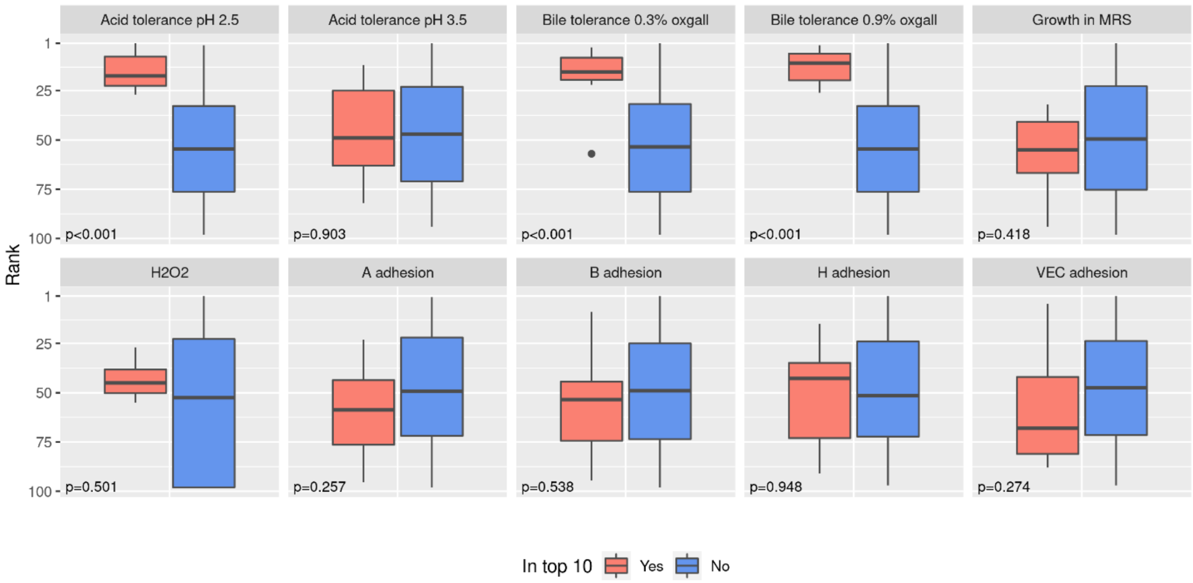

Acid tolerance pH 2.5
Acid tolerance pH 3.5
Bile tolerance 0.3% oxgall
Bile tolerance 0.9% oxgall
Growth in MRS
H2O2
A adhesion
B adhesion
H adhesion
VEC adhesion
Rank
p<0.001
p=0.903
p<0.001
p<0.001
p=0.418
p=0.501
p=0.257
p=0.538
p=0.948
p=0.274
In top 10
Yes
No

9B)

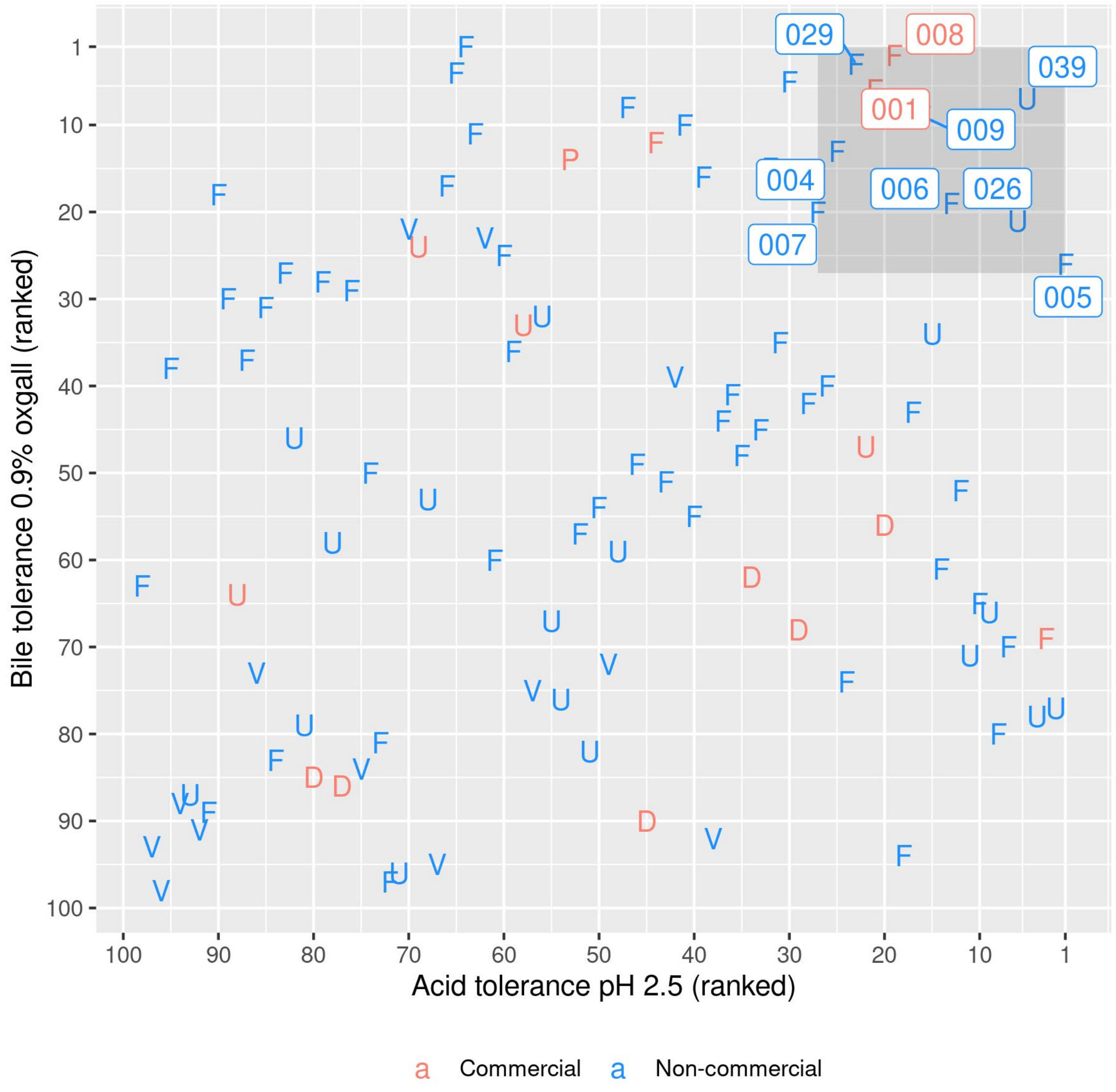

Bile tolerance 0.9% oxgall (ranked)
Acid tolerance pH 2.5 (ranked)
029
008
039
001
009
004
006
026
007
005
Commercial
Non-commercial

Supplementary figure 9. A) Characteristics of the 10 best performing strains with rank-ordered acid (pH 2.5) and strong bile (0.9 % Oxgall) tolerances in comparison to the other strains in the dataset. The box shows the interquartile range, the line inside the box indicates the median, whiskers the minimum and the maximum values, and dots represent the outliers. B) Data projection of rank-ordered strong acid tolerance (pH 2.5) vs bile tolerance (0.9 % Oxgall) in two dimensions. The top right corner indicates 10 strains that perform the best in terms of these parameters. Different letters indicate the different sources of strains F= Fecal, D = dairy, P= plant, V = vagina, U = unknown and colors whether the strain is commercial (red) or non-commercial (blue). The numbers indicating the strains are depicted on Table 1.

10A

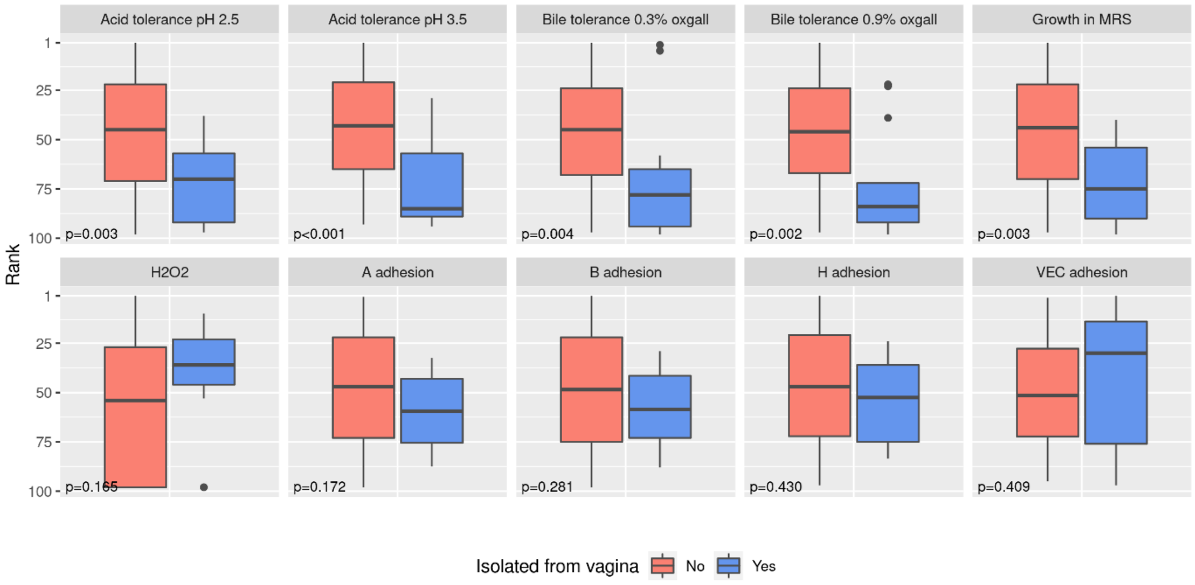

10B)

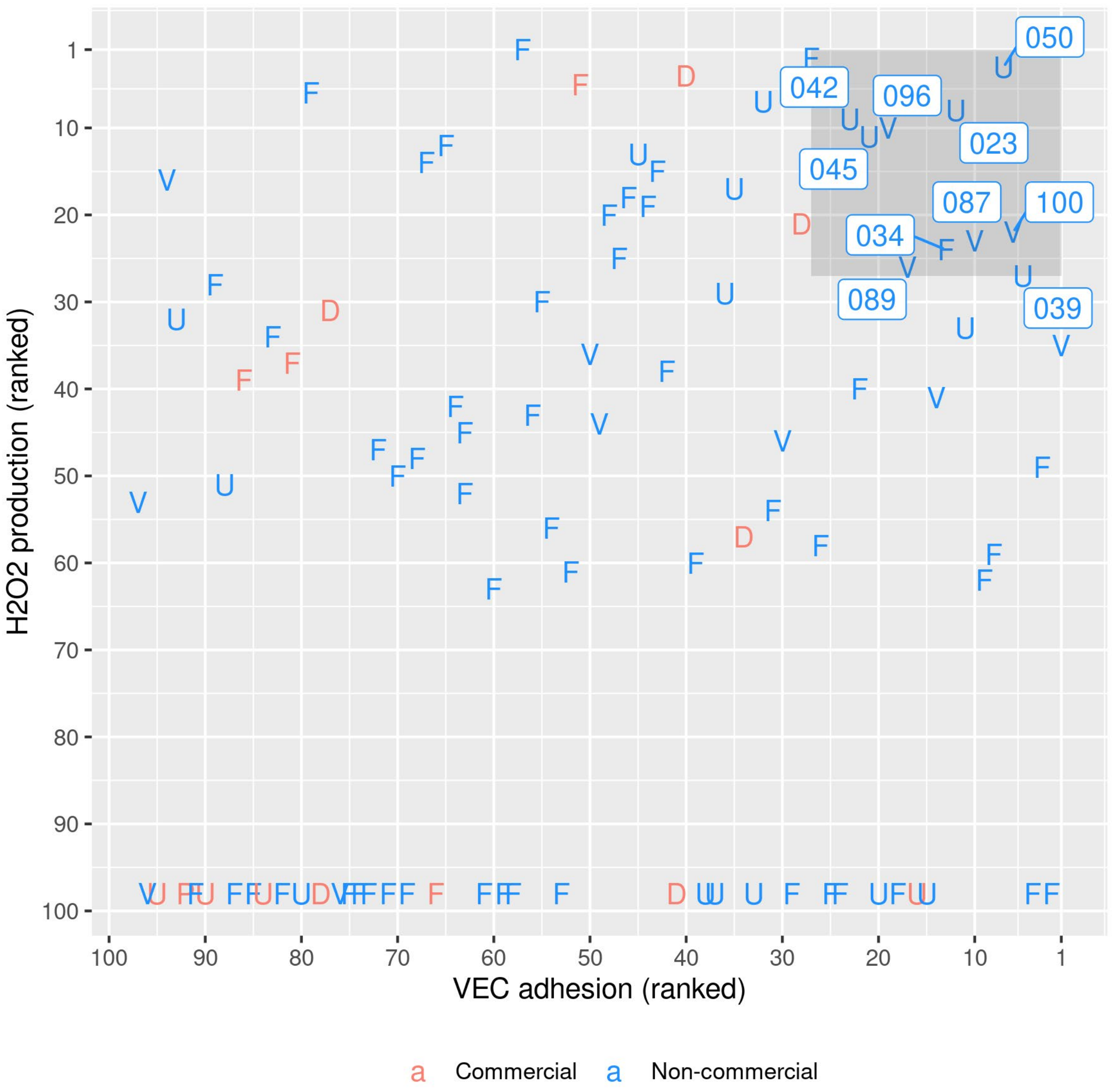

H2O2 production (ranked)
VEC adhesion (ranked)
050
042
096
023
045
087
100
034
089
039
Commercial
Non-commercial

10C)

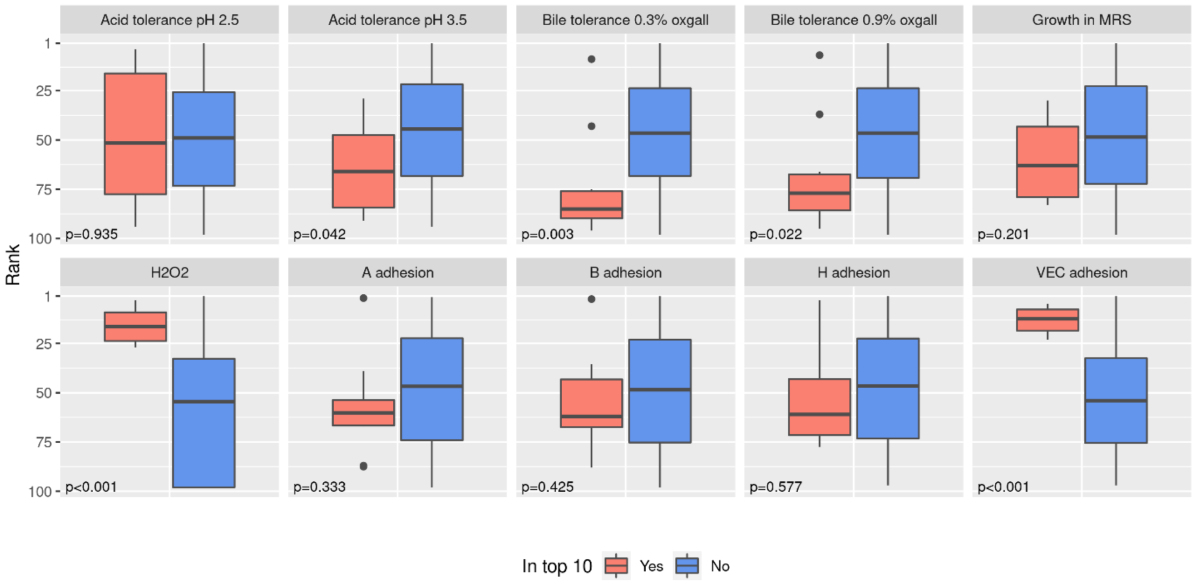

Supplementary figure 10. Characteristics of the 13 vaginally isolated lactobacilli (blue bars, rank ordered data) in comparison to strains from other origin (red bars, rank ordered data). A) Data projection of rank-ordered vaginal epithelial cell adhesion to H_2O_2 production in two dimensions. B) From the top right corner indicates a set of 10 strains that performed the best in terms of these attributes. C) Characteristics of the ten best performing strains with rank-ordered vaginal cell adhesion and hydrogen peroxide production in comparison to the other strains in the dataset. Different letters indicate the different sources of strains F= Fecal, D = dairy, P= plant, V = vagina, U = unknown and colors whether the strain is commercial (red) or non-commercial (blue). The numbers indicating the strains are depicted on Table 1.

Supplementary Tables

Table S1. Statistical analysis of growth in MRS OD600. Probiotic growth in MRS at species level. Two *L. iners* strains from the panel did not grow, and were omitted from the analysis. Species comparison using Welch t-test, row vs column. Red color means that row is statistically significantly higher than the column, blue vice versa. Cells show the estimated difference in means above the FDR-adjusted t-test p-value. The FDR-adjustment was obtained using the Benjamini-Hochberg procedure.

Species	*L. brevis* (n=3)	*L. paracasei* (n=7)	*L. rhamnosus* (n=20)	*L. crispatus* (n=9)	*L. fermentum* (n=10)	*L. gasseri* (n=6)	*L. jensenii* (n=6)	*L. plantarum* (n=5)	*L. iners* (n=3)
L. acidophilus (n=14)	0.08<br>0.238	-0.05<br>0.365	-0.05<br>0.369	0.03<br>0.736	0.05<br>0.501	0.11<br>0.445	0.04<br>0.471	-0.11<br>0.196	0.42<br>0.008
L. brevis (n=3)		-0.13<br>0.118	-0.13<br>0.114	-0.05<br>0.663	-0.02<br>0.783	0.03<br>0.810	-0.04<br>0.580	-0.19<br>0.084	0.34<br>0.014
L. paracasei (n=7)			0<br>0.986	0.08<br>0.445	0.11<br>0.250	0.16<br>0.289	0.09<br>0.202	-0.06<br>0.475	0.47<br><0.001
L. rhamnosus (n=20)				0.08<br>0.446	0.11<br>0.252	0.16<br>0.289	0.09<br>0.202	-0.06<br>0.490	0.47<br><0.001
L. crispatus (n=9)					0.02<br>0.853	0.08<br>0.631	0.01<br>0.943	-0.14<br>0.237	0.39<br>0.006
L. fermentum (n=10)						0.06<br>0.701	-0.01<br>0.876	-0.17<br>0.132	0.36<br>0.003
L. gasseri (n=6)							-0.07<br>0.627	-0.22<br>0.185	0.31<br>0.089
L. jensenii (n=6)								-0.15<br>0.121	0.38<br>0.003
L. plantarum (n=5)									0.53<br>0.002

Table S2. Statistical analysis of acid tolerance pH 3.5, log reduction. Acid tolerance at species level at pH 3.5. Two *L. iners* strains from the panel did not grow, and were omitted from the analysis. Data was missing from *L. acidophilus* LA11897, *L. amylovorus* LX11898, *L. paracasei* LC11896, and *L. ruminis* LX11894, and these were omitted from the analysis as well. Species comparison using Welch t-test, row vs column. Red color means that row is statistically significantly higher than the column, blue vice versa. Cells show the estimated difference in means above the FDR-adjusted t-test p-value. The FDR-adjustment was obtained using the Benjamini-Hochberg procedure.

Species	*L. brevis* (n=3)	*L. paracasei* (n=7)	*L. rhamnosus* (n=20)	*L. crispatus* (n=9)	*L. fermentum* (n=10)	*L. gasseri* (n=6)	*L. jensenii* (n=6)	*L. plantarum* (n=5)	*L. iners* (n=3)
L. acidophilus (n=14)	0.09<br>0.619	-0.24<br>0.130	-0.21<br>0.033	0.24<br>0.430	-0.32<br>0.066	-0.13<br>0.197	1.26<br>0.087	0.24<br>0.251	0.56<br>0.196
L. brevis (n=3)		-0.33<br>0.174	-0.3<br>0.199	0.15<br>0.633	-0.41<br>0.117	-0.22<br>0.293	1.18<br>0.102	0.15<br>0.518	0.47<br>0.226
L. paracasei (n=7)			0.03<br>0.825	0.48<br>0.155	-0.08<br>0.653	0.11<br>0.437	1.5<br>0.052	0.48<br>0.073	0.79<br>0.115
L. rhamnosus (n=20)				0.45<br>0.167	-0.11<br>0.480	0.08<br>0.379	1.48<br>0.057	0.45<br>0.084	0.77<br>0.132
L. crispatus (n=9)					-0.56<br>0.117	-0.37<br>0.237	1.03<br>0.132	0<br>0.986	0.32<br>0.429
L. fermentum (n=10)						0.19<br>0.227	1.58<br>0.043	0.56<br>0.042	0.87<br>0.087
L. gasseri (n=6)							1.39<br>0.067	0.37<br>0.124	0.68<br>0.151
L. jensenii (n=6)								-1.02<br>0.130	-0.71<br>0.262
L. plantarum (n=5)									0.31<br>0.374

Table S3. Statistical analysis of acid tolerance pH 2.5, log reduction. Acid tolerance at species level at pH 2.5. Two *L. iners* strains from the panel did not grow, and were omitted from the analysis. Species comparison using Welch t-test, row vs column. Red color means that row is statistically significantly than the column, blue vice versa. Cells show the estimated difference in means above the FDR-adjusted t-test p-value. The FDR-adjustment was obtained using the Benjamini-Hochberg procedure.

Species	*L. brevis* (n=3)	*L. paracasei* (n=7)	*L. rhamnosus* (n=20)	*L. crispatus* (n=9)	*L.* fermentum (n=10)	*L. gasseri* (n=6)	*L. jensenii* (n=6)	*L. plantarum* (n=5)	*L. iners* (n=3)
L. acidophilus (n=14)	2.19<br>0.073	0.3<br>0.518	0.54<br>0.130	1.59<br>0.001	1.07<br>0.132	-1.65<br><0.001	2.31<br>0.011	1.24<br>0.007	1.01<br>0.121
L. brevis (n=3)		-1.89<br>0.084	-1.65<br>0.129	-0.61<br>0.418	-1.12<br>0.213	-3.85<br>0.039	0.12<br>0.885	-0.95<br>0.250	-1.18<br>0.196
L. paracasei (n=7)			0.24<br>0.579	1.28<br>0.026	0.77<br>0.288	-1.96<br>0.003	2<br>0.022	0.94<br>0.086	0.71<br>0.242
L. rhamnosus (n=20)				1.04<br>0.014	0.52<br>0.415	-2.2<br><0.001	1.76<br>0.036	0.7<br>0.073	0.47<br>0.369
L. crispatus (n=9)					-0.52<br>0.445	-3.24<br><0.001	0.72<br>0.289	-0.34<br>0.404	-0.57<br>0.297
L. fermentum (n=10)						-2.72<br>0.002	1.24<br>0.155	0.17<br>0.783	-0.06<br>0.936
L. gasseri (n=6)							3.96<br>0.002	2.89<br><0.001	2.66<br>0.025
L. jensenii (n=6)								-1.07<br>0.141	-1.3<br>0.118
L. plantarum (n=5)									-0.23<br>0.631

Table S4. Statistical analysis of bile tolerance, growth in 0.3% oxgall % of growth in MRS w/o bile. Bile tolerance in 0.3 % oxgall compared to growth in MRS without bile (oxgall). Two *L. iners* strains from the panel did not grow and were omitted from the analysis completely. Species comparison using Welch t-test, row vs column. Red color means that row is statistically significantly higher than the column, blue vice versa. Cells show the estimated difference in means above the FDR-adjusted t-test p-value. The FDR-adjustment was obtained using the Benjamini-Hochberg procedure.

Species	*L. brevis* (n=3)	*L. paracasei* (n=7)	*L. rhamnosus* (n=20)	*L. crispatus* (n=9)	*L. fermentum* (n=10)	*L. gasseri* (n=6)	*L. jensenii* (n=6)	*L. plantarum* (n=5)	*L. iners* (n=3)
L. acidophilus (n=14)	9.6<br>0.430	35.7<br>0.001	33.4<br><0.001	57<br><0.001	19<br>0.117	31<br>0.141	83.3<br><0.001	0.4<br>0.955	-1.1<br>0.966
L. brevis (n=3)		26.1<br>0.118	23.8<br>0.147	47.4<br>0.018	9.5<br>0.505	21.4<br>0.296	73.7<br>0.040	-9.2<br>0.460	-10.7<br>0.687
L. paracasei (n=7)			-2.3<br>0.754	21.4<br>0.085	-16.6<br>0.191	-4.7<br>0.786	47.6<br><0.001	-35.3<br>0.004	-36.8<br>0.288
L. rhamnosus (n=20)				23.7<br>0.039	-14.3<br>0.204	-2.4<br>0.888	49.9<br><0.001	-33<br>0.003	-34.5<br>0.307
L. crispatus (n=9)					-38<br>0.012	-26.1<br>0.204	26.3<br>0.025	-56.6<br><0.001	-58.1<br>0.162
L. fermentum (n=10)						11.9<br>0.539	64.3<br><0.001	-18.7<br>0.141	-20.1<br>0.498
L. gasseri (n=6)							52.3<br>0.042	-30.6<br>0.147	-32.1<br>0.340
L. jensenii (n=6)								-82.9<br><0.001	-84.4<br>0.121
L. plantarum (n=5)									-1.5<br>0.955

Table S5. Statistical analysis of bile tolerance, growth in 0.9% oxgall % of growth in MRS w/o bile. Bile tolerance in 0.9 % oxgall compared to growth in MRS without bile (oxgall). Two *L. iners* strains from the panel did not grow and were omitted from the analysis completely. Species comparison using Welch t-test, row vs column. Red color means that row is statistically significantly higher than the column, blue vice versa. Cells show the estimated difference in means above the FDR-adjusted t-test p-value. The FDR-adjustment was obtained using the Benjamini-Hochberg procedure.

Species	*L. brevis* (n=3)	*L. paracasei* (n=7)	*L. rhamnosus* (n=20)	*L. crispatus* (n=9)	*L. fermentum* (n=10)	*L. gasseri* (n=6)	*L. jensenii* (n=6)	*L. plantarum* (n=5)	*L. iners* (n=3)
L. acidophilus (n=14)	50.7<br>0.067	77.5<br><0.001	77.3<br><0.001	97.1<br><0.001	66.2<br><0.001	93.3<br><0.001	104<br><0.001	17.8<br>0.369	38.5<br>0.211
L. brevis (n=3)		26.8<br>0.175	26.5<br>0.196	46.4<br>0.087	15.5<br>0.369	42.6<br>0.118	53.3<br>0.093	-32.9<br>0.184	-12.3<br>0.622
L. paracasei (n=7)			-0.2<br>0.972	19.6<br>0.052	-11.3<br>0.354	15.8<br>0.090	26.5<br>0.015	-59.7<br>0.035	-39<br>0.203
L. rhamnosus (n=20)				19.8<br>0.004	-11<br>0.289	16.1<br><0.001	26.7<br><0.001	-59.4<br>0.042	-38.8<br>0.216
L. crispatus (n=9)					-30.9<br>0.014	-3.7<br>0.487	6.9<br>0.202	-79.3<br>0.016	-58.6<br>0.132
L. fermentum (n=10)						27.1<br>0.025	37.8<br>0.005	-48.4<br>0.062	-27.7<br>0.302
L. gasseri (n=6)							10.6<br>0.006	-75.5<br>0.024	-54.9<br>0.152
L. jensenii (n=6)								-86.2<br>0.016	-65.5<br>0.128
L. plantarum (n=5)									20.7<br>0.460

Table S6. Statistical analysis of hydrogen peroxide production at 3 hours % of comparator strain. Hydrogen peroxide (H_2O_2) production compared to comparator strain *L. jensenii* DSM 20557 at 3 h time point. Two *L. iners* strains from the panel did not grow and were omitted from the analysis completely. *L. brevis* Lbr-35, *L. brevis* LX11860, *L. casei* Lc-11, *L. curvatus* 360, *L. fermentum* SBS-1, *L. fermentum 238, L. fermentum* 2342, *L. fermentum* 1924, *L. gasseri* Lg-36, *L. iners* Li21-23, *L. iners* Li14-7, *L. johnsonii* LG0883, *L. rhamnosus* LX11870, *L. rhamnosus* LC11868, *L. rhamnosus* LX11882, *L. rhamnosus* Lr-32, *L. rhamnosus* LX11877, *L. rhamnosus* LX11879, *L. rhamnosus* LX11862, *L. rhamnosus* LX11857, *L. rhamnosus* 1704, *L. rhamnosus* LX11889, *L. rhamnosus* LR1049, *L. salivarius* Ls-33, all *L. paracasei* and all *L. plantarum* strains did not produce H_2O_2 at all, and were omitted from the analysis. Species comparison using Welch t-test, row vs column. Red color means that row is statistically significantly higher than the column, blue vice versa. Cells show the estimated difference in means above the FDR-adjusted t-test p-value. The FDR-adjustment was obtained using the Benjamini-Hochberg procedure.

Species	*L. brevis* (n=3)	*L. paracasei* (n=7)	*L. rhamnosus* (n=20)	*L. crispatus* (n=9)	*L. fermentum* (n=10)	*L. gasseri* (n=6)	*L. jensenii* (n=6)	*L. plantarum* (n=5)	*L. iners* (n=3)
L. acidophilus (n=14)	72.5<br>0.036	85.9<br><0.001	80.7<br><0.001	-36.1<br>0.281	18.1<br>0.579	12.8<br>0.653	-100.1<br>0.054	85.9<br><0.001	78<br>0.002
L. brevis (n=3)		13.4<br>0.523	8.3<br>0.672	-108.6<br>0.012	-54.4<br>0.141	-59.7<br>0.112	-172.5<br>0.007	13.4<br>0.523	5.6<br>0.787
L. paracasei (n=7)			-5.2<br>0.030	-122<br>0.004	-67.8<br>0.052	-73.1<br>0.046	-185.9<br>0.008		-7.9<br>0.523
L. rhamnosus (n=20)				-116.9<br>0.005	-62.6<br>0.070	-67.9<br>0.057	-180.8<br>0.009	5.2<br>0.030	-2.7<br>0.808
L. crispatus (n=9)					54.2<br>0.194	48.9<br>0.202	-63.9<br>0.196	122<br>0.004	114.2<br>0.005
L. fermentum (n=10)						-5.3<br>0.885	-118.1<br>0.033	67.8<br>0.052	59.9<br>0.087
L. gasseri (n=6)							-112.8<br>0.039	73.1<br>0.046	65.2<br>0.062
L. jensenii (n=6)								185.9<br>0.008	178.1<br>0.007
L. plantarum (n=5)									-7.9<br>0.523

Table S7. Statistical analysis of epithelial cell adhesion % of adhesion of comparator. Adhesion to vaginal epithelial cells in comparison to comparator strain *L. jensenii* LX11796. Two *L. iners* strains from the panel did not grow and were omitted from the analysis completely. Species comparison using Welch t-test, row vs column. Red color means that row is statistically significantly higher than the column, blue vice versa. Cells show the estimated difference in means above the FDR-adjusted t-test p-value. The FDR-adjustment was obtained using the Benjamini-Hochberg procedure.

Species	*L. brevis* (n=3)	*L. paracasei* (n=7)	*L. rhamnosus* (n=20)	*L. crispatus* (n=9)	*L. fermentum* (n=10)	*L. gasseri* (n=6)	*L. jensenii* (n=6)	*L. plantarum* (n=5)	*L. iners* (n=3)
L. acidophilus (n=14)	-99.6<br>0.185	-17.8<br>0.580	-13.9<br>0.121	-46.1<br>0.175	-6.6<br>0.529	-15.9<br>0.300	-36.4<br>0.202	-5.9<br>0.683	41.3<br>0.251
L. brevis (n=3)		81.8<br>0.199	85.7<br>0.204	53.5<br>0.358	93<br>0.191	83.7<br>0.202	63.2<br>0.283	93.6<br>0.182	140.9<br>0.093
L. paracasei (n=7)			3.9<br>0.905	-28.3<br>0.512	11.2<br>0.723	1.9<br>0.955	-18.7<br>0.631	11.8<br>0.719	59<br>0.184
L. rhamnosus (n=20)				-32.2<br>0.319	7.3<br>0.563	-2<br>0.899	-22.5<br>0.407	8<br>0.631	55.2<br>0.175
L. crispatus (n=9)					39.4<br>0.237	30.2<br>0.373	9.6<br>0.788	40.1<br>0.247	87.3<br>0.066
L. fermentum (n=10)						-9.3<br>0.580	-29.8<br>0.289	0.7<br>0.968	47.9<br>0.199
L. gasseri (n=6)							-20.5<br>0.473	10<br>0.619	57.2<br>0.146
L. jensenii (n=6)								30.5<br>0.302	77.7<br>0.087
L. plantarum (n=5)									47.2<br>0.197

Table S8. Statistical analysis of average A antigen adhesion % of adhesion of comparator. Average A antigen adhesion, % of adhesion of *L. crispatus* LMG 18204. Two *L. iners* strains from the panel did not grow and were omitted from the analysis completely. Species comparison using Welch t-test, row vs column. Red color means that row is statistically significantly stat. sig. higher than the column, blue vice versa. Cells show the estimated difference in means above the FDR-adjusted t-test p-value. The FDR-adjustment was obtained using the Benjamini-Hochberg procedure.

Species	*L. brevis* (n=3)	*L. paracasei* (n=7)	*L. rhamnosus* (n=20)	*L. crispatus* (n=9)	*L. fermentum* (n=10)	*L. gasseri* (n=6)	*L. jensenii* (n=6)	*L. plantarum* (n=5)	*L. iners* (n=3)
L. acidophilus (n=14)	-7.9<br>0.683	-10.8<br>0.651	-17.2<br>0.075	10.5<br>0.181	-39.9<br>0.207	12.1<br>0.121	13.6<br>0.087	33.4<br>0.523	12.7<br>0.116
L. brevis (n=3)		-2.9<br>0.920	-9.4<br>0.634	18.4<br>0.415	-32.1<br>0.364	20<br>0.384	21.5<br>0.365	41.3<br>0.460	20.6<br>0.374
L. paracasei (n=7)			-6.4<br>0.782	21.3<br>0.387	-29.1<br>0.437	22.9<br>0.360	24.4<br>0.325	44.2<br>0.445	23.5<br>0.348
L. rhamnosus (n=20)				27.7<br><0.001	-22.7<br>0.456	29.4<br><0.001	30.9<br><0.001	50.6<br>0.369	29.9<br><0.001
L. crispatus (n=9)					-50.5<br>0.130	1.6<br>0.556	3.1<br>0.227	22.9<br>0.651	2.2<br>0.450
L. fermentum (n=10)						52.1<br>0.121	53.6<br>0.117	73.4<br>0.240	52.6<br>0.119
L. gasseri (n=6)							1.5<br>0.370	21.3<br>0.670	0.6<br>0.786
L. jensenii (n=6)								19.8<br>0.685	-0.9<br>0.605
L. plantarum (n=5)									-20.7<br>0.673

Table S9. Statistical analysis of average B antigen adhesion % of adhesion of comparator. Average B antigen adhesion, % of adhesion of *L. crispatus* LMG 18204. Two *L. iners* strains from the panel did not grow and were omitted from the analysis completely. Species comparison using Welch t-test, row vs column. Red color means that row is statistically significantly higher than the column, blue vice versa. Cells show the estimated difference in means above the FDR-adjusted t-test p-value. The FDR-adjustment was obtained using the Benjamini-Hochberg procedure.

Species	*L. brevis* (n=3)	*L. paracasei* (n=7)	*L. rhamnosus* (n=20)	*L. crispatus* (n=9)	*L. fermentum* (n=10)	*L. gasseri* (n=6)	*L. jensenii* (n=6)	*L. plantarum* (n=5)	*L. iners* (n=3)
L. acidophilus (n=14)	-7.8<br>0.269	-5.6<br>0.487	-11.5<br>0.142	4.7<br>0.407	-41.9<br>0.167	3.9<br>0.512	8<br>0.147	29.6<br>0.580	7.1<br>0.202
L. brevis (n=3)		2.1<br>0.777	-3.7<br>0.603	12.5<br>0.117	-34.1<br>0.237	11.7<br>0.119	15.8<br>0.114	37.4<br>0.498	14.9<br>0.103
L. paracasei (n=7)			-5.8<br>0.490	10.3<br>0.175	-36.2<br>0.213	9.6<br>0.205	13.7<br>0.096	35.2<br>0.518	12.8<br>0.116
L. rhamnosus (n=20)				16.2<br>0.014	-30.4<br>0.289	15.4<br>0.028	19.5<br>0.003	41.1<br>0.461	18.6<br>0.005
L. crispatus (n=9)					-46.5<br>0.130	-0.8<br>0.833	3.3<br>0.202	24.9<br>0.633	2.5<br>0.407
L. fermentum (n=10)						45.8<br>0.132	49.9<br>0.116	71.5<br>0.256	49<br>0.118
L. gasseri (n=6)							4.1<br>0.260	25.7<br>0.631	3.2<br>0.394
L. jensenii (n=6)								21.6<br>0.672	-0.9<br>0.659
L. plantarum (n=5)									-22.5<br>0.663

Table S10. Statistical analysis of average H antigen adhesion % of adhesion of comparator. Average H antigen adhesion, % of adhesion of *L. crispatus* LMG 18204. Two *L. iners* strains from the panel did not grow and were omitted from the analysis completely. Species comparison using Welch t-test, row vs column. Red color means that row is statistically significantly higher than the column, blue vice versa. Cells show the estimated difference in means above the FDR-adjusted t-test p-value. The FDR-adjustment was obtained using the Benjamini-Hochberg procedure.

Species	*L. brevis* (n=3)	*L. paracasei* (n=7)	*L. rhamnosus* (n=20)	*L. crispatus* (n=9)	*L. fermentum* (n=10)	*L. gasseri* (n=6)	*L. jensenii* (n=6)	*L. plantarum* (n=5)	*L. iners* (n=3)
L. acidophilus (n=14)	-0.7<br>0.943	11.3<br>0.191	-9<br>0.202	4<br>0.501	-41.4<br>0.172	4.2<br>0.487	7.4<br>0.199	26.4<br>0.631	6.3<br>0.272
L. brevis (n=3)		12<br>0.247	-8.4<br>0.367	4.7<br>0.563	-40.7<br>0.180	4.9<br>0.546	8<br>0.374	27<br>0.627	6.9<br>0.429
L. paracasei (n=7)			-20.3<br>0.025	-7.3<br>0.297	-52.7<br>0.102	-7.1<br>0.318	-3.9<br>0.529	15<br>0.778	-5<br>0.445
L. rhamnosus (n=20)				13<br>0.014	-32.4<br>0.256	13.3<br>0.017	16.4<br>0.001	35.4<br>0.523	15.3<br>0.003
L. crispatus (n=9)					-45.4<br>0.133	0.2<br>0.952	3.4<br>0.213	22.4<br>0.672	2.3<br>0.429
L. fermentum (n=10)						45.6<br>0.132	48.8<br>0.118	67.8<br>0.289	47.7<br>0.121
L. gasseri (n=6)							3.1<br>0.310	22.1<br>0.673	2<br>0.515
L. jensenii (n=6)								19<br>0.716	-1.1<br>0.475
L. plantarum (n=5)									-20.1<br>0.699
